# Using Random Regression Models to Genetically Evaluate Functional Longevity Traits in North American Angus Cattle

**DOI:** 10.3390/ani10122410

**Published:** 2020-12-16

**Authors:** Hinayah R. Oliveira, Luiz F. Brito, Stephen P. Miller, Flavio S. Schenkel

**Affiliations:** 1 Centre for Genetic Improvement of Livestock, Department of Animal Biosciences, University of Guelph, Guelph, ON N1G 2W1, Canada; schenkel@uoguelph.ca; 2 Department of Animal Sciences, Purdue University, West Lafayette, IN 47907, USA; britol@purdue.edu; 3 Angus Genetics Inc., Saint Joseph, MO 64506, USA; SMiller@angus.org

**Keywords:** beef cattle, lifespan, longitudinal trait, productive life, stayability, survival

## Abstract

**Simple Summary:**

Cattle longevity is usually defined as the duration of life of a cow from first calving to death. In addition to a longer lifespan, it is crucial that cows are productive throughout their lives. Incorporating optimal indicators of productive longevity in breeding schemes will directly improve the economic profitability of the beef cattle herd and long-term sustainability of the industry. Thus, the impact of different longevity indicators in the selection of North American Angus cattle was evaluated and optimal parameters were defined to perform the evaluations.

**Abstract:**

This study aimed to propose novel longevity indicators by comparing genetic parameters for traditional (TL; i.e., the cow’s lifespan after the first calving) and functional (FL; i.e., how long the cow stayed in the herd while also calving; assuming no missing (FLa) or missing (FLb) records for unknown calving) longevity, considering different culling reasons (natural death, structural problems, disease, fertility, performance, and miscellaneous). Longevity definitions were evaluated from 2 to 15 years of age, using single- and multiple-trait Bayesian random regression models (RRM). The RRM fitting heterogenous residual variance and fourth order Legendre polynomials were considered as the optimal models for the majority of longevity indicators. The average heritability estimates over ages for FLb (from 0.08 to 0.25) were always higher than those for FLa (from 0.07 to 0.19), and higher or equal to the ones estimated for TL (from 0.07 to 0.23), considering the different culling reasons. The average genetic correlations estimated between ages were low to moderate (~0.40), for all longevity definitions and culling reasons. However, removing the extreme ages (i.e., 2 and >12 years) increased the average correlation between ages (from ~0.40 to >0.70). The genetic correlations estimated between culling reasons were low (0.12 and 0.20 on average, considering all ages and ages between 3 and 12 years old, respectively), indicating that longevity based on different culling reasons should be considered as different traits in the genetic evaluations. Higher average genetic correlations (estimated from 3 to 12 years old) were observed between TL and FLb (0.73) in comparison to TL and FLa (0.64), or FLa and FLb (0.65). Consequently, a higher average proportion of commonly-selected sires, for the top 1% sires, was also observed between TL and FLb (91.74%), compared to TL and FLa (59.68%), or FLa and FLb (61.01%). Higher prediction accuracies for the expected daughter performances (calculated based on the pedigree information) were obtained for FLb in comparison to TL and FLa. Our findings indicate that FLb is preferred for the genetic evaluation of longevity. In addition, it is recommended including multiple longevity traits based on different groups of culling reasons in a selection sub-index, as they are genetically-different traits. Genetic selection based on breeding values at the age of four years is expected to result in greater selection responses for increased longevity in North American Angus cattle.

## 1. Introduction

The profitability and long-term sustainability of the beef cattle industry is dependent on several factors, including feed-related variables (e.g., feed costs, animal feed efficiency), environmental footprints of the industry, disease resilience, and climatic adaptation. In the United States, the top beef cattle producer in the world [1], longevity has been identified by beef cattle stakeholders in a nation-wide survey as the utmost priority for further improvement [2]. Longevity is of paramount importance for the beef cattle industry because it directly impacts the economic return in any beef cattle production system [3,4]. Improving beef cattle longevity will increase the overall farm profitability by both decreasing the costs associated with rearing heifers, and increasing the number of productive mature animals in the herd [3,5]. Moreover, longevity is an indirect indicator of animal health, resilience, and welfare [6,7,8].

Longevity is usually defined as the duration of life of a cow from first calving to natural death [6,9,10]. However, in addition to a longer lifespan, it is crucial that the animals are productive throughout their lives. Therefore, several other definitions of longevity have been proposed over time. For instance, Brzáková et al. [11] recently defined longevity as the period of time from the first to the last calving, while Ramos et al. [12] defined longevity based on the number of calvings until a certain age. In addition, stayability to consecutive calvings [13] or the probability that a cow remained in the herd until a specific age, given it calved once [14] have also been proposed as indicators of cow longevity. However, despite the wide number of definitions available, there is still a lack of studies comparing their use in genetic evaluations of longevity, especially when considering specific culling reasons.

Regardless the trait definition, longevity relies on voluntary and involuntary culling performed by individual farmers [9,15]. In general, voluntary culling depends on the animals’ productivity and temperament, while involuntary culling is a result of several factors such as disease, reproductive disorders, structural problems, and natural death. Culling many animals due to involuntary reasons can hamper the genetic progress in a population and, consequently, be detrimental to the breeding program [15]. Moreover, culling reasons can be associated to multiple biological functions and underlying genetic mechanisms, which are reflected in the estimation of variance components used in genetic and genomic evaluations. However, to our best knowledge, there are no studies evaluating genetic parameters for multiple longevity indicators considering the specific culling reasons. In this context, distinguishing the impact of each culling reason in the estimation of variance components and genetic parameters for longevity traits can greatly impact the performance of genetic and genomic evaluations for longevity.

Various statistical models have been used to genetically evaluate longevity in cattle. For instance, Jamrozik et al. [16] compared the use of proportional hazard, multiple-trait, and random regression models (RRM) to evaluate what the authors defined as functional survival (i.e., time of culling after the first calving), using a cattle simulated dataset. The authors observed that the predictive ability of RRM was greater compared to other mentioned models, indicating that RRM is the method of choice for genetically evaluating longevity-related traits in cattle. Furthermore, Sánchez-Castro et al. [17] showed that linear RRM have a better predictive performance for stayability (analyzed as the probability that a cow will remain in the herd until 6 years old) compared to traditional threshold models. Nonetheless, as suggested by Corrales et al. [18] and Oliveira et al. [19], the predictive performance of RRM relies on the polynomial order chosen to model the fixed and random regressions, as well as the adequate definition of the number of classes for the residual variance, which are usually population and trait specific.

The overarching goal of this study was to evaluate the feasibility of RRM to genetically evaluate longevity in North American Angus cattle. The specific objectives were to: (a) define the best time-periods to perform selection for longevity in North American Angus cattle; (b) evaluate the impact of different longevity definitions (i.e., traditional and functional longevity) for genetic selection; (c) investigate the impact of different culling reasons in the estimation of variance components and genetic parameters for longevity indicators; and (d) propose an optimal RRM to genetically evaluate longevity in North American Angus cattle, in terms of polynomial order and number of classes for the residual variance.

## 2. Materials and Methods

### 2.1. Ethics Statement

No animal care committee approval was necessary for the purposes of this study, as all information required was extracted from existing databases obtained in accordance with standard farm management practices of commercial breeders.

### 2.2. Datasets, Culling Reasons, and Quality Control

The American Angus Association (Saint Joseph, MO, USA) and the Canadian Angus Association (Rocky View County, AB, Canada) provided the pedigree, calving, and culling datasets used in this study. First, a phenotypic quality control was performed to remove records from cows with missing birth date, herd identification, and culling reason or date. Records from cows with more than one culling date and/or more than one culling reason reported in the dataset were also removed. In addition, cows born before 1990 or that did not have their first calving before 30 months of age were excluded from the dataset. The minimum age considered for the first calving was 19 months, while the maximum culling age was 20 years. After the phenotypic quality control, data from 527,283 and 77,260 cows from USA and Canada, respectively, were used to create the longevity traits. Preliminary analyses indicated that data from USA and Canada could be combined in the same genetic evaluation (results not shown).

To evaluate the impact of different culling reasons on the estimation of variance components and genetic parameters, a total of seven different groups of culling reasons were created: (1) natural death, which included cows that were culled over 15 years old and cows that died naturally due to unknown reasons; (2) structural problems, which included cows that were culled due to body structure, teat and udder conformation, feet conformation, eye problem, and rectal or vaginal prolapse; (3) disease, which included cows culled due to different illnesses or diseases; (4) fertility, which included cows culled due to fertility related problems and that did not become pregnant in a breeding season; (5) performance, which included cows culled due to own or progeny productivity performance for growth and carcass traits, or temperament; (6) miscellaneous, which included cows culled before 15 years old without specific reasons and cows sold as commercial; and (7) all reasons, which combined all the previously mentioned culling groups. Details about the number of animals included in each culling group (after the quality control) are shown in Table 1.

**Figure 1:**
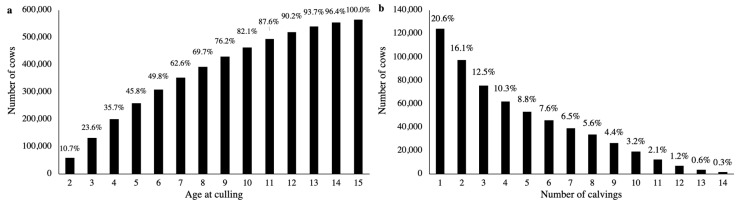
Number of cows by culling age (cumulative; (**a**)) and by number of calvings (**b**). Age at culling equal to 15 included cows culled from 15 to 20 years-old.

Relatives of animals with phenotypic records from up to 10 generations back were included in the pedigree files. After the quality control and pedigree pruning, the number of animals included in the pedigree was 796,090 for the group of natural death, 375,416 for structural problems, 153,743 for disease, 883,143 for fertility, 561,000 for performance, 1,005,644 for miscellaneous, and 1,631,390 considering all reasons together. The distribution of the number of cows by culling age and number of calvings are shown in Figure 1. The distribution of the number of cows by culling age and number of calvings within each group of culling reason are shown in the Figures S1 and S2 (in the Supplementary Material).

### 2.3. Longevity Definitions

In order to evaluate the impact of different longevity indicators in Angus breeding programs, two definitions were used in this study:

**Traditional longevity (TL).** TL was defined as the time from first calving to culling. Thus, a total of 14 binary records were assigned to each cow (from 2 to 15 years old), for cows that had their first calving between 19 to 30 months old. The binary records for each specific age of each cow were coded as 1 (when the cow was alive) and 0 (after culling).

**Functional longevity (FL).** FL was defined as the time period in which the cow was alive and calving after its first calving. Thus, the 14 binary records (from 2 to 15 years old) assigned to each cow considered if the cow had calved or not. Two different scenarios were used to code the binary records for FL: (a) records were codified as 1 for cows that calved at the specific age, and 0 after the cow was culled or if she did not record a calf at the specific age (FLa); and (b) records were codified as 1 for cows that calved at the specific age, 0 after the cow was culled, and as missing records when no information of calving was found at the specific age (FLb).

The proportion of 0, 1, and missing values over time for each longevity definition are shown in Figure S3 (Supplementary Material).

### 2.4. Statistical Analyses

**Methods.** Bayesian methods based on the Gibbs sampler and Markov Chain Monte Carlo (MCMC) algorithm were used to estimate the variance components for all groups of culling reason and longevity indicators. A MCMC chain length of 500,000 cycles, considering a burn-in period of 250,000 and a sampling interval (thin) of 10 cycles were used. Convergence was verified using the Heidelberger and Welch [20] and Geweke [21] criteria, both available in the package “*boa—Bayesian Output Analysis*” [22] of the R software [23]. Variance component estimation and breeding value prediction (EBVs) were performed using the gibbs3f90 software [24].

**Single-trait models.** Initially, all groups of culling reason and longevity definitions were analyzed through Bayesian single-trait RRM. In matrix notation, the RRM used are defined as follow:

y=Xb+Hq+Za+Wp+e,

in which **y** is the vector of observations, **b** is the vector of systematic effects, **q** is the vector of random regression coefficients for herd-year-season effects, **a** is the vector of random regression coefficients for animal additive genetic effects, **p** is the vector of random regression coefficients for permanent environmental effects, and **e** is the vector of random residuals. The **X**, **H**, **Z**, and **W** are the incidence matrices for **b**, **q**, **a**, and **p**, respectively. The systematic effects included in **b** are embryo transfer (i.e., if the cow was born through embryo transfer; assumed as a categorical variable), and the coefficients of systematic regressions for year-season of birth (i.e., average regression for each year-season of birth). Second, third, and fourth order Legendre orthogonal polynomials [25] were tested for the regressions (see details in the *“Model comparison”* section).

It was assumed that $y|b,q,a,p,\;R_{q},\;G_{0},R_{p},\sigma_{e\left(x\right)}^{2}∼N\left(\mathrm{Xb}+\mathrm{Hq}+\mathrm{Za}+\mathrm{Wp},\;I\sigma_{e\left(x\right)}^{2}\right)$, in which $R_{q},\;G_{0},\;\mathrm{and}\;R_{p}$ are the herd-year-season, additive genetic, and permanent environmental (co)variance components matrices for the random regression coefficients, respectively. The $\sigma_{e\left(x\right)}^{2}$ is the residual variance, which was tested to be homogeneous or heterogenous with 14 different classes (one class for each age). Thus, $\sigma_{e\left(x\right)}^{2}$ was assumed as $\sigma_{e\left(x\right)}^{2}=\sigma_{e}^{2}$ for homogeneity of variances, and $\sigma_{e\left(x\right)}^{2}=\sigma_{\mathrm{ej}}^{2}$ for heterogeneity (i.e., j = 2, 3, …, 15). For **b**, it was assumed that $b∼N\left(0,\Sigma_{b}⨂I\right)$, in which $\Sigma_{b}$ is a diagonal matrix with large variances (10^10^) to represent vague prior knowledge, and I is an identity matrix. The **q**, **a**, and **p** were assumed as $q|R_{q}∼N\left(0,R_{q}⨂I\right)$, $a|G_{0},\;A∼N\left(0,G_{0}⨂A\right)$, and $p|R_{p}∼N\left(0,R_{p}⨂I\right)$, respectively, in which **A** is the pedigree-based relationship matrix, and all other parameters were previously defined. The $R_{q},\;G_{0},\;\mathrm{and}\;R_{p}$ were assumed to follow an inverted Wishart distribution (IW) with small prior knowledge, i.e., $R_{q}∼\mathrm{IW}\left(3,{\hat{R}}_{q}\right)$, $G_{0}∼\mathrm{IW}\left(3,{\hat{G}}_{0}\right)$, and $R_{q}∼\mathrm{IW}\left(3,{\hat{R}}_{q}\right)$, where ${\hat{R}}_{q},{\hat{G}}_{0}$, and ${\hat{R}}_{q}$ are the estimated (co)variance matrices, and a scaled inverted chi-squared distribution was assumed for $\sigma_{e\left(x\right)}^{2}$.

**Model comparison.** As previously mentioned, second, third, and fourth order Legendre orthogonal polynomials [25] were tested to describe the systematic (i.e., year-season of birth), and random (i.e., herd-year-season, additive genetic and the permanent environmental) regressions for each group of culling reason and longevity definition. In order to reduce the number of comparisons and keep the model parsimony, the same polynomial order was used to describe all curves, as suggested by Jamrozik et al. [13]. Thus, a general notation to represent the tested models is given by LEG2, LEG3, and LEG4, when using second, third, or fourth polynomial order, respectively. Furthermore, two alternative scenarios were tested for the residual variance after defining the optimal polynomial order: homogeneous (the same variance was assumed for all ages), and heterogeneous (one variance was assumed for each age, from 2 to 15 years) residual variance. Polynomial orders were tested using homogeneous residual variance, as suboptimal polynomial orders can make the residuals heterogeneous just because of the lack of fitting.

All tested models for each group of culling reason and longevity definition were compared using the Deviance Information Criterion (DIC; [26]), calculated as follow:

$$\mathrm{DIC}=D\left(\bar{\theta }\right)+2p_{D}$$

in which $D\left(\bar{\theta }\right)$ is the deviance obtained by replacing the parameters by their posterior mean estimates in the likelihood function, and $2p_{D}$ is the effective number of parameters used in the model. In order to facilitate the comparison based on DIC values, the posterior model probability (PMP) was also calculated [27,28]. The PMP is defined as:

$$p(M_{s}|y)=\frac{\exp\left(\Delta_{s}/2\right)}{\sum_{s=1}^{S}\exp\left(\Delta_{s}/2\right)}$$

in which $p(M_{s}|y)$ is the posterior probability of the model “*s*” to be the best model; $\exp\left(\Delta_{s}/2\right)$ is the exponential of the DIC difference between model “s” and the best model (i.e., the model with the lowest DIC); and $\sum_{s=1}^{S}\exp\left(\Delta_{s}/2\right)$ is the summation of the exponential of the DIC differences from all tested models, i.e., from the first “s” to the last “S” model. The model with higher PMP is preferred to describe the data.

**Multiple-trait model.** In order to better understand the genetic relationship between the different culling reasons, Bayesian multiple-trait RRM were used. Thus, known culling reasons (i.e., natural death, structural problems, disease, fertility, and performance) were assumed as different traits in a five-trait RRM (total of three analysis, one for each longevity definition). The total number of animals with phenotypes in these multiple-trait analyses was 396,451, and a total of 1,117,640 animals were included in the pedigree file (up to 10 generations back from the phenotyped animals). Similarly, multiple-trait RRM were also used to evaluate the genetic relationship between the different longevity indicators (i.e., TL, FLa, and FLb). In this case, five analyses (one for each known culling reason) were performed using three-trait RRM. The number of animals included in the pedigree and phenotypic file for these three-trait RRM are equal to the ones previously described in the topic *“Datasets, culling reasons, and quality control”*.

Multiple-trait RRM considered the optimal polynomial order, systematic and random effects, and number of residual variance classes defined in the single-trait model comparisons. The same systematic and random effects previously mentioned were included in the statistical model, and the same parameters were used for the MCMC chain (i.e., 500,000, 250,000 and 10, for chain length, burn-in, and thin, respectively). In addition, similar statistical assumptions were made for both single- and multiple-trait RRM.

### 2.5. Estimation of Variance Components, Genetic Parameters, and EBVs over Time

**Variance components**. The herd-year-season, additive genetic, and permanent environmental (co)variances for all analyzed ages were calculated using the posterior mean of the (co)variance components estimated for the random regression coefficients of these effects, i.e.,:

$$\varphi =TR_{q}T^{\prime },\;\sum =TG_{0}T^{\prime },\;\mathrm{and}\;\theta =TR_{p}T^{\prime },$$

where φ, ∑, and θ are the herd-year-season, additive genetic, and permanent environmental (co)variance matrices for the ages, respectively, and **T** is a matrix of independent covariates for the optimal Legendre polynomial order defined in this study and adapted according to the number of traits analyzed for the multiple-trait analyses. The $R_{q},\;G_{0},\;\mathrm{and}\;R_{p}$ are the previously mentioned herd-year-season, additive genetic, and permanent environmental (co)variance components matrices for the random regression coefficients, which contain the posterior means obtained from the posterior marginal distribution samples for each effect (after burn-in and thin). Residual variances ($\sigma_{e\left(x\right)}^{2}$) were also obtained from the posterior marginal distribution samples.

**Genetic parameters.** Heritabilities were calculated using the variance components estimated based on the optimal RRM defined in the single-trait analyses, for each group of culling reasons and longevity definitions. Thus, the heritabilities over the different ages for each group of culling reasons and longevity definitions were obtained as:

$${\hat{h}}_{j}^{2}=\frac{{\hat{\sigma }}_{a_{j}}^{2}}{{\hat{\sigma }}_{a_{j}}^{2}+{\hat{\sigma }}_{q_{j}}^{2}+{\hat{\sigma }}_{p_{j}}^{2}+{\hat{\sigma }}_{e_{\left(x\right)}}^{2}},$$

in which ${\hat{h}}_{j}^{2}$ is the heritability estimated for the age j (j = 2, 3, …, 15), ${\hat{\sigma }}_{a_{j}}^{2},\;{\hat{\sigma }}_{q_{j}}^{2},\;\mathrm{and}\;{\hat{\sigma }}_{p_{j}}^{2}$ are the additive genetic, herd-year-season, and permanent environmental variances estimated for the age j (found in the jth diagonal of the φ, ∑, and θ matrices, respectively), and ${\hat{\sigma }}_{e_{\left(x\right)}}^{2}$ is the estimated residual variance, which depends on the approach used (i.e., homogeneous or heterogeneous).

Genetic correlations estimated across different ages under the same longevity definition and group of culling reason were obtained as:

$${\hat{r}}_{{\mathrm{jj}}^{\prime }}=\frac{{\hat{\sigma }}_{a_{{\mathrm{jj}}^{\prime }}}}{\sqrt{{\hat{\sigma }}_{a_{j}}^{2}{\hat{\sigma }}_{a_{j^{\prime }}}^{2}}}$$

in which ${\hat{r}}_{{\mathrm{jj}}^{\prime }}$ is the genetic correlation estimated between the age j and j′; ${\hat{\sigma }}_{a_{{\mathrm{jj}}^{\prime }}}$ is the additive genetic covariances estimated between the j and j′ ages; and ${\hat{\sigma }}_{a_{j}}^{2}$ and ${\hat{\sigma }}_{a_{j^{\prime }}}^{2}$ are the additive genetic variances estimated for the ages j and j′, respectively. Similarly, the genetic correlation across ages between different groups of culling reason and longevity definitions were obtained as:

$${\hat{r}}_{c_{j}c_{j}^{\prime }}=\frac{{\hat{\sigma }}_{a_{c_{j}c_{j}^{\prime }}}}{\sqrt{{\hat{\sigma }}_{a_{c_{j}}}^{2}{\hat{\sigma }}_{a_{c_{j}^{\prime }}}^{2}}}\;\mathrm{and}\;{\hat{r}}_{t_{j}t_{j}^{\prime }}=\frac{{\hat{\sigma }}_{a_{t_{j}t_{j}^{\prime }}}}{\sqrt{{\hat{\sigma }}_{a_{t_{j}}}^{2}{\hat{\sigma }}_{a_{t_{j}^{\prime }}}^{2}}},$$

in which ${\hat{r}}_{c_{j}c_{j}^{\prime }}$ and ${\hat{r}}_{t_{j}t_{j}^{\prime }}$ are the genetic correlations estimated across ages between the different groups of culling reason and longevity definitions, respectively; ${\hat{\sigma }}_{a_{c_{j}c_{j}^{\prime }}}$ and ${\hat{\sigma }}_{a_{t_{j}t_{j}^{\prime }}}$ are the additive genetic covariances estimated across ages between the different groups of culling reason and longevity definitions, respectively; ${\hat{\sigma }}_{a_{c_{j}}}^{2}\;\mathrm{and}\;{\hat{\sigma }}_{a_{c_{j}^{\prime }}}^{2}$ are the additive genetic variances estimated for the first and second culling reason, respectively; and ${\hat{\sigma }}_{a_{t_{j}}}^{2}\;\mathrm{and}\;{\hat{\sigma }}_{a_{t_{j}^{\prime }}}^{2}$ are the additive genetic variances estimated for the first and second longevity definition, respectively.

Genetic correlations estimated across ages under the same longevity definition and group of culling reason were obtained from the single-trait analyses, and genetic correlations estimated among groups of culling reason and longevity definitions were obtained from the multiple-trait analyses (using the five- and three-trait RRM, respectively). Genetic correlations were calculated for all known culling reasons (i.e., natural death, structural problems, disease, fertility, and performance).

**EBVs.** Breeding values for all different ages of the animal **i**, for each group of culling reason and longevity definition, were obtained as:

$${\mathrm{EBV}}_{i}=T{\hat{a}}_{i},$$

in which ${\mathrm{EBV}}_{i}$ is the vector of breeding values for the animal **i** that includes all analyzed ages, ${\hat{a}}_{i}$ is the vector of breeding values for the regression coefficients of the animal **i**, and **T** is the previously mentioned matrix of independent covariates associated with the Legendre polynomial, adapted according to the number of traits.

### 2.6. Impact of Longevity Definition in the Selection Scheme

In order to facilitate the comparison of the impact of different longevity definitions in the breeding program, the proportion of animals commonly selected, based on their EBVs for each age, was calculated. The proportion of commonly selected animals was calculated considering the top 1% and 10% sires with more than 5 daughters with longevity records in the dataset. The number of sires used for the group of natural death, structural problems, disease, fertility, and performance was 1285, 1297, 523, 1471, and 1616, respectively. The EBVs used for the comparisons were predicted using three-trait RRM (i.e., considering TL, FLa, and FLb as different traits; total of five analyses, one for each culling reason). Additionally, EBVs for each sire were translated into the expected daughter longevity (EDL) using a linear regression of the EDL on the relative breeding values (RBV), i.e.,

$$\mathrm{EDL}=b_{0}+b_{1}\times \mathrm{RBV},$$

where $b_{0}$ and $b_{1}$ were previously calculated using the daughter performance observed in the dataset, and the RBV are the sires’ EBV standardized to scale that has average of 100 and standard deviation of 5 [29]. In summary, RBV were used in this study in order to simplify the understanding and maintain consistency among proof expression of different longevity indicators. The RBV estimated from 2 to 6 years-old were used to predict four different EDL: the daughter’s average culling age and the proportion of daughters alive at 6, 9, and 12 years old. Prediction accuracy was calculated as the Pearson correlation coefficient between expected and observed daughter longevity.

## 3. Results

### 3.1. Descriptive Statistics

The highest proportion of animals analyzed in this study was from the group where culling reason was missing a specific reason (i.e., miscellaneous; ~34%), followed by the group of fertility (~26%) and natural death (~25%). Only a small proportion of animals died due to disease (approximately 1%) or were removed due to structural problems (~4%). In addition, approximately 10% of the animals in the dataset were culled due to voluntary reasons (i.e., due to performance; Table 1).

In general, animals from the group of natural death tended to die older than animals culled for other reasons. On the other hand, animals culled due to fertility related problems tended to die younger than animals culled due to other reasons (Figure S1, Supplementary Material). The average (SD) culling age was 9.75 (3.43), 6.85 (2.90), 5.48 (2.88), 4.33 (2.97), 5.02 (2.87), and 4.91 (3.52) years, for the group of natural death, structural problems, disease, fertility, performance, and miscellaneous, respectively. When considering all groups of culling reason together, approximately 90% of the animals in the dataset were culled before 12 years old. The proportion of cows that had more than 10 calvings was small (~7%), and about 20% of the cows had only one calving reported in the dataset (Figure 1).

### 3.2. Model Comparison

For all groups of culling reason and longevity definitions, LEG4 (i.e., RRM based on fourth order Legendre orthogonal polynomials to describe the average, herd-year-season, additive genetic, and permanent environmental effects) outperformed the models fitting lower polynomial orders and, therefore, they were considered as optimal RRM when assuming homogeneity of residual variance. The DIC and PMP values estimated for the different polynomial orders using homogeneous residual variance, for each group of culling reason and longevity definition, are shown in Table S1 (Supplementary Material). The DIC and PMP values estimated for the RRM using LEG4 and homogeneity or heterogeneity of residual variance, for each group of culling reason and longevity definition, are shown in Table 2.

In summary, the RRM including heterogeneity of residual variance yielded a better fit for all groups of culling reason when considering both FLa and FLb. However, when analyzing TL, the RRM considering heterogeneity of residual variance did not outperform the RRM considering homogeneity of residual variance for two groups of culling reason: structural problems and disease (Table 2). The differences observed in the number of classes of the residual variance for these two groups of culling reason compared to the other ones are likely related to the smaller number of phenotypic records available for them (Table 1).

**Figure 2:**
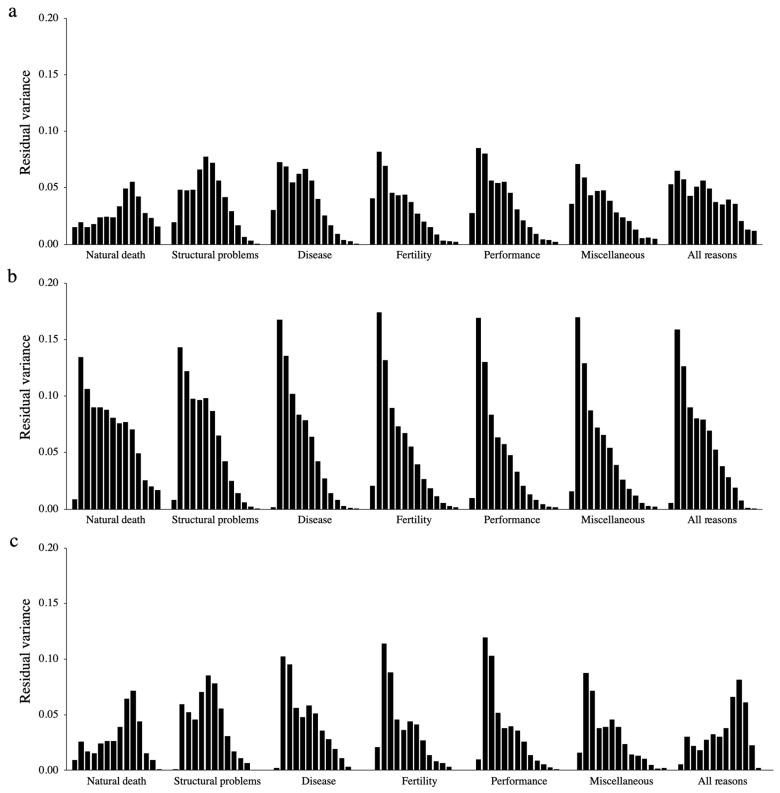
Residual variance estimated across different ages (from 2 to 15 years), considering all groups of culling reason (i.e., natural death, structural problems, disease, fertility, performance, miscellaneous, and all culling reasons together) and longevity definitions. The longevity definitions are: (**a**) traditional longevity, (**b**) functional longevity assuming 0 after the cow was culled or if the cow did not record a calf at the specified age, and (**c**) functional longevity assuming 0 only after the cow was culled, and missing records when no information of calving was found at the specific age.

Higher residual variances were estimated for FLa compared to TL and FLb. In addition, for the majority of culling reasons and longevity definitions, greater residual variances were estimated at 3 and 4 years, and smaller residual variances were estimated in the extremes of the age interval (2 and >12 years). However, TL and FLb had higher residual variances between 8 and 11 years for the culling group of natural death, and between 5 and 8 years for the culling group of structural problems. The residual variances estimated at different ages, considering all groups of culling reason and longevity definitions, are shown in Figure 2.

The differences in the residual variances estimated at different ages within each group of culling reason and longevity definition corroborate with the conclusions drawn from the DIC and PMP criteria, which indicated the need for using heterogeneous residual variance in most scenarios. Thus, due to the strong evidences that RRM based on LEG4 using heterogeneous residual variance are the most suitable models for genetically evaluating longevity considering the majority of groups of culling reasons and longevity definitions analyzed in this study, detailed results will only be presented for this model. Estimates from LEG4 using homogeneous residual variance will be provided for comparison purposes when relevant.

### 3.3. Genetic Parameters

**Figure 3:**
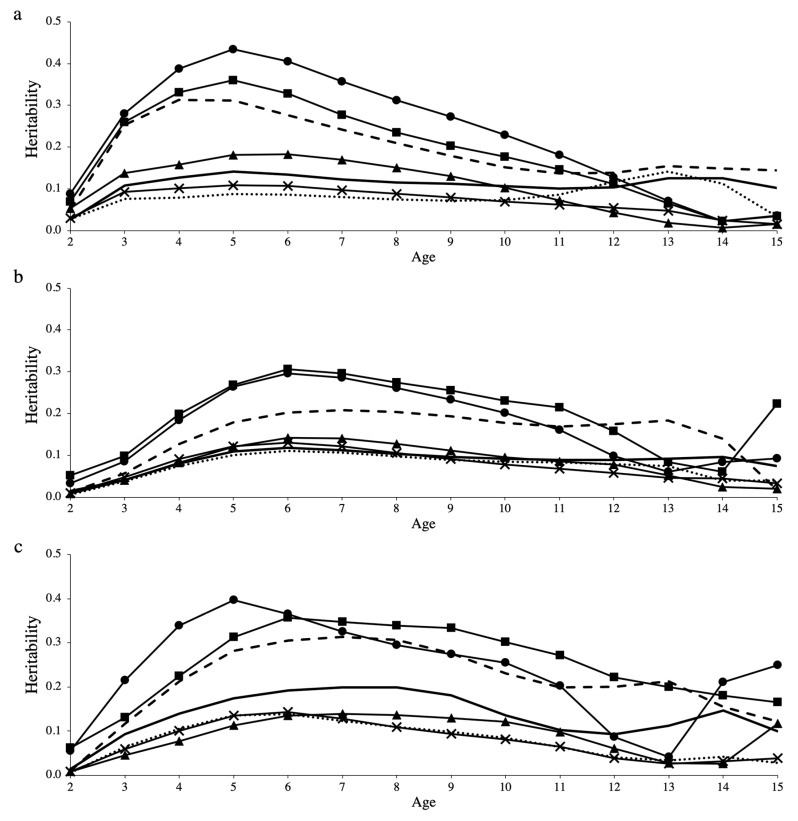
Heritabilities estimated over the different ages, for all groups of culling reason and longevity definitions, using heterogeneous residual variance. The groups of culling reasons are: natural death (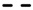), structural problems (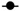), disease (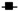), fertility (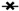), performance (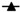), miscellaneous (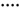), and all reasons together (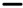). The longevity definitions are: (**a**) traditional longevity, (**b**) functional longevity assuming 0 after the cow was culled or if the cow did not record a calf at the specific age, and (**c**) functional longevity assuming 0 only after the cow was culled, and missing records when no information of calving was found for the specific age.

**Heritabilities.** Heritabilities estimated using LEG4 under heterogeneous residual variance, for all groups of culling reason and longevity definitions, are shown in Figure 3. Heritabilities estimated using LEG4 under homogeneous residual variance are shown in Figure S4 (Supplementary Material). In general, similar patterns of heritability estimates over time were observed using either homogeneity or heterogeneity of residual variance, for the majority of culling reasons and longevity definitions.

For all longevity definitions, the highest heritability estimates were obtained for the animals that died due to structural problems and disease. On the other hand, the lowest heritabilities were estimated for animals that died due to performance, fertility, and without information (i.e., miscellaneous). In addition, the pattern of heritabilities estimated when combining all culling reasons was, in general, similar to the culling groups of performance, fertility, and miscellaneous. The heritability estimates for the culling group of natural death tended to be higher than those for the culling groups of fertility, performance, and miscellaneous, and lower than the estimates for the groups of structural problems and disease.

In general, the peaks of heritability observed for FLa occurred in latter ages compared to TL (for most cases FLa peaks occurred between 5 to 7 years, while for TL they occurred between 4 to 6 years). However, for FLb a greater variability in the heritability patterns was observed, as the ages in which the peaks of heritability occurred ranged according to the culling reason analyzed. For the majority of groups of culling reasons in all longevity definitions, lower heritabilities were obtained at 2 and over 12 years compared to the intermediate ages. However, there was a sudden increase in the heritability estimates observed at high ages for the group of disease under the FLa definition (Figure 3b), and the groups of structural problems and performance under the FLb definition (Figure 3c). When disregarding the culling reasons, the average heritability estimates for FLb were always higher compared to those for FLa, and either higher or equal to the average heritabilities estimated for TL. The average heritability estimates when considering all ages and ages between 3 and 12 years old, for all longevity definitions and culling reasons, are shown in Table 3.

**Genetic correlations.** For clarity, the genetic correlations are presented in three sections: (1) between ages; (2) between culling reasons; and (3) between longevity definitions.

#### 3.3.1. Genetic Correlations between Ages

**Figure 4:**
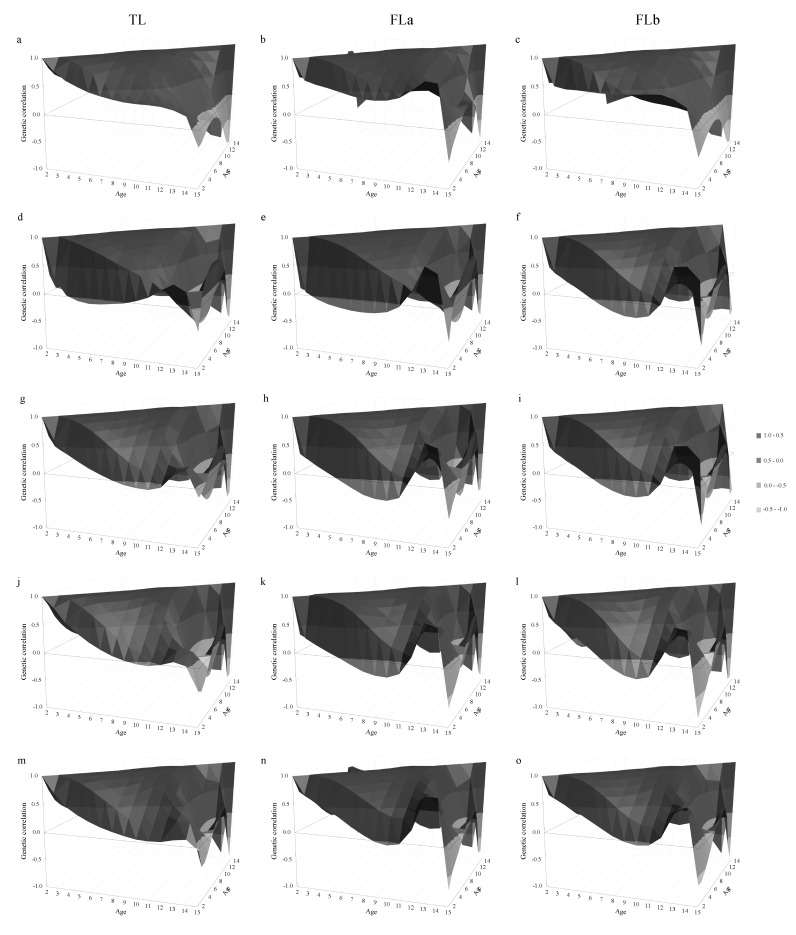
Genetic correlations estimated across ages, for all groups of known culling reason and longevity definitions. The groups of culling reason are: natural death (**a**–**c**); structural problems (**d**–**f**); disease (**g**–**i**); fertility (**j**–**l**); and performance (**m**–**o**). The longevity definitions are: traditional longevity (TL); functional longevity assuming 0 after the cow was culled or if the cow did not record a calf at the specified age (FL_a_); and functional longevity assuming 0 only after the cow was culled, and missing records when no information of calving was found at the specific age (FL_b_), respectively.

Genetic correlations estimated across ages, for all groups of culling reasons and longevity definitions, are shown in Figure 4. In general, similar patterns of genetic correlations were observed across longevity definitions within the same group of culling reason. High genetic correlations (above 0.90) were observed for adjacent age groups, especially for ages between 3 and 12 years. However, the magnitude of correlations decreased with increasing gap between ages. Negative genetic correlations were found mainly for ages greater than 12 years. The average genetic correlations estimated considering all ages and ages between 3 and 12 years, for all longevity definitions and culling reasons are shown in Table 4.

In general, average genetic correlations had low to moderate magnitude (~0.40) when considering all ages together. This indicates that longevity evaluated at different ages can be considered as genetically different traits, especially for ages further apart. However, as expected, removing the extreme ages (2 and >12 years) increased the average correlation for all longevity definitions and culling reasons. In this case, the average genetic correlations were higher (greater than 0.70), indicating that longevity evaluated at different ages within this interval are good indicators of longevity up to 12 years-old. The groups of natural death and performance had higher average genetic correlations between ages compared to the other groups of culling reasons. Among the different longevity definitions, FLa tended to have slightly higher average genetic correlations compared to TL and FLb (Table 4).

#### 3.3.2. Genetic Correlations between Culling Reasons

The average genetic correlations estimated between the different culling reasons over ages, for all longevity definitions, are shown in Table 5. Genetic correlations estimated between culling reasons were low (0.12 and 0.20 average considering all ages and ages between 3 and 12 years, respectively), indicating that longevity based on different culling reasons should be considered as different traits in genetic and genomic evaluations. Among the pairs of culling reasons, the highest genetic correlations were found between the groups of animals culled due fertility and performance (0.24 and 0.39 average, considering all ages and ages between 3 and 12 years, respectively). Moreover, higher genetic correlations between culling reasons tended to be found for FLa.

#### 3.3.3. Genetic Correlations between Longevity Definitions

Higher genetic correlations were observed between TL and FLb compared to between TL and FLa, and between FLa and FLb. For all trait comparisons, higher genetic correlations were observed at closer ages, especially between 3 to 12 years. Lower genetic correlations were observed for the culling group of fertility for all comparisons of longevity definitions. Average genetic correlations between the different longevity definitions over ages, for all groups of known culling reason, are shown in Table 6. Genetic correlations estimated between the different longevity definitions over ages, for all groups of culling reason, are shown in Figure S8 (Supplementary Material).

### 3.4. Impact of Longevity Definition on the Selection Scheme

The proportion of commonly-selected sires when considering the different longevity definitions are shown in Table 7. As expected, a higher proportion of animals selected in common was found between TL and FLb, compared to TL and FLa, or FLa and FLb. Even when selecting the top 10% bulls, no proportion was higher than 90%, indicating that different selection decisions would be made depending on the longevity definition used in the breeding programs.

The average prediction accuracy of the EDL between the different groups of known culling reasons, considering all longevity definitions and ages at selection, are shown in Table 8. Prediction accuracy of the daughter’s average culling age calculated within each group of known culling reason are shown in Figure S9 (Supplementary Material). Prediction accuracy of the proportion of daughters alive at 6, 9, and 12 years-old, calculated within each group of known culling reason, are shown in Figure S10 (Supplementary Material). In general, the average prediction accuracy increased with greater ages at selection. Similar average prediction accuracies were calculated for daughters’ average culling age and proportion of daughters alive at 6 years. The lowest accuracies were obtained when predicting EDL for proportion of daughters alive at 12 years, followed by the proportion of daughters alive at 9 years. In most cases, FLb tended to yield slightly higher accuracies compared to TL and FLa (except for EDL predicted for proportion of daughters alive at 12 years, in which FLa yielded the highest accuracy). Nonetheless, the prediction accuracies estimated ranged from low to moderate (i.e., from 0.05 to 0.30). The highest average improvement in accuracy compared to previous age categories was found when the age at selection was 3 years old (for proportion of daughters alive at 6 and 9 years), and 4 years (for daughter’s average culling age and proportion of daughters alive at 12 years).

Average EDL predicted for the top and bottom 1% and 10% sires for TL, FLa, and FLb, considering all groups of known culling reasons and selection at 4 years old, are shown in Table 9, Table 10 and Table 11, respectively. The average difference between the top and bottom sires calculated for all ages at selection analyzed (i.e., from 2 to 6 years), considering all groups of known culling reasons and longevity definitions, are shown in Tables S2–S4 (Supplementary Material). In summary, more similar average EDL was predicted between TL and FLb compared to FLa. However, the dispersion of EDL tended to be greater for FLb compared to both TL and FLa (i.e., larger standard deviations were found for FLb). As expected, the proportion of daughters alive decreased with age, and the highest and lowest daughters’ average culling age were predicted for the groups of natural death and fertility, respectively. Average difference between the top and bottom sires tended to be greater for the culling group of performance and smaller for the group of natural death. In addition, no specific pattern between top and bottom sires was observed between longevity definitions.

## 4. Discussion

### 4.1. Descriptive Statistics

Cow longevity is a very complex and important trait in breeding programs [3,4,30]. In general, the economic profitability of cattle production increases with an increase in longevity, as most decisions on culling of cows are based on their productivity instead of involuntary reasons [4,31,32]. However, the improvement of longevity through genetic selection is suboptimal due to several factors. First, the late expression of the phenotype (i.e., end of life) considerably increases the generation interval, which reduces genetic progress per time unit. Moreover, the lack of information regarding the culling reasons can potentially generate bias in the genetic and genomic evaluations, as some animals do not have the opportunity to fully express their genetic merit for longevity. In order to evaluate the impact of different culling reasons in the estimation of genetic parameters for longevity and avoid the interference of censored data in the results, only animals that had culling information were kept in this study. Nonetheless, we recognize the need for evaluating the impact of censored data in subsequent studies for genetic analyses of longevity.

As suggested by Jamrozik et al. [13], decisions on removal of a cow from the herd may involve several reasons and not all of them are usually reported in the dataset. Most animals analyzed in this study were from the culling group of miscellaneous, which included both cows culled before 15 years of age without specific reasons and cows sold as commercial animals (Table 1). Thus, as suggested by Rózańska-Zawieja et al. [15], it is advisable to provide more specific culling reasons in the farm recording programs. Only a small proportion of animals were culled due to disease and structural problems, which suggests that the methods used for prevention and control of diseases, as well as selection against structural problems (e.g., feet conformation), have been effective in the North American Angus population. For both the American Angus Association [33] and Canadian Angus Association [34], expected progeny differences (EPDs) are predicted for claw set and foot angle in a joint analyses. Selecting for adequate claw set and foot angle can reduce the incidence of lameness [35], one of the main reasons for early culling in beef cattle [36,37]. This in agreement with Vargas et al. [38], who commented that locomotion disorders can lead to several productive and reproductive losses.

The high proportion of animals culled due to fertility-related issues found in this study is in agreement with Rózańska-Zawieja et al. [15], who reported that reproductive disorders were the most common culling reason for animals from Brahman, Hereford, and Angus breeds raised in Poland. Similarly, Koeck et al. [39] reported that about 25% of Holstein cows were culled due to reproductive problems. Our study also indicated that cows with fertility issues were culled at a younger age than cows culled due to other reasons, as was also reported by Morales et al. [40]. In this context, Burris and Priode [41] showed that cows calving late in a breeding season are usually culled sooner. Moreover, Cushman et al. [42] and Damiran et al. [32] showed that this feature is even stronger for heifers, i.e., heifers that calve later at their first calving fail to remain in the herd as long as heifers that calve earlier (first 21 days in the breeding season). Furthermore, discarding cows that did not become pregnant in a breeding season is a reasonable strategy used by several farmers to reduce future economic losses [3].

The number of calvings per cow found in our study (Figure 1) is in agreement with Brzáková et al. [11], who found that 22% of the beef cows from Czech Republic had only one calving. Animals from the group of natural death tended to die older than animals from the other groups, however, the average culling age found in our study for the group of natural death (9.75 years) was lower than the average reported by Rózańska-Zawieja et al. [15] for beef cattle from United States and Canada (i.e., 12.70 and 13.00 years, respectively). Only ~10% of the animals included in this study were culled due to performance, which reinforces the importance of genetically evaluating longevity in North American Angus cattle (Table 1).

### 4.2. Model Comparison

The statistical models used can influence the predictive performance of EBVs. Therefore, model definition is a crucial step in genetic and genomic evaluations. Longevity traits were first evaluated using non-linear proportional hazard models [43,44]. In summary, hazard models allow to easily account for censored data (i.e., animals without culling information), and the inclusion of time-dependent environmental effects [44]. However, the main disadvantage of this model is the fact that it only allows the estimation of a single genetic effect for each animal during its whole life [43]. Thus, in order to avoid the complexity of hazard models and predict EBVs for all ages, Veerkamp et al. [43] proposed that a RRM can be used for genetic analyses of longevity related traits. Nowadays, RRM seems to be the optimal choice to genetically evaluate longevity over time [16,17,19]. The predictive performance of RRM relies on how well the model fits the data, which is strongly related to the type and order of polynomials used [18,19]. Several studies have reported that Legendre orthogonal polynomials are preferred for genetic analysis of a variety of traits compared to other types of polynomials [45,46]. However, the polynomial order considered as optimal tends to be population and trait specific.

Especially for longevity-related traits, different orders of Legendre polynomials have been assumed as optimal. For instance, third order Legendre polynomials were chosen to genetically evaluate stayability to consecutive calvings in Canadian Simmental [13]. On the other hand, Plaengkaeo et al. [47], who tested different Legendre polynomial orders to evaluate longevity in swine, concluded that second order Legendre polynomial should be used for genetic evaluations. Moreover, Haile-Mariam and Pryce [48] found that fitting only the intercept was more adequate to analyze longevity in Australian Holstein cattle. However, the mentioned authors decided to use first order Legendre polynomials in order to study the association between longevity and other traits (such as production, fertility, and type traits) over time [48]. In our study, three different polynomial orders were evaluated (i.e., LEG2, LEG3, and LEG4) and the more parameterized models (LEG4) outperformed the simpler models for all groups of culling reason and longevity definitions (Supplementary Table S1). Thus, the improvement in the quality of the fit seems to compensate for the increase in the models’ complexity for genetic analysis of longevity in North American Angus cattle.

Assuming homogeneity of residual variance might not be realistic for genetic analysis of longevity, as it indicates that the variance due to non-explained effects remains constant over time. For this reason, after choosing the optimal polynomial order, LEG4 models assuming homogeneity and heterogeneity of residual variance were compared. Testing the polynomial order before the number of classes used for the residual variance is a common practice reported in the scientific literature [49]. As expected, using heterogeneous residual variance improved the model’s fit for all groups of culling reason when considering FL as the analyzed trait. However, for TL, RRM using heterogeneity of residual variance did not improve the model’s fit in two different groups of culling reason: structural problems and disease, which might be related to the smaller number of observations for these groups and the consequent increase in models’ complexity when using heterogeneous residual variance. In this context, especially for TL, the smaller number of observations might have reduced the phenotypic variability in adjacent ages, as records assumed for a specific age are likely more related to the previous age when using TL compared to FLa and FLb (i.e., TL does not consider calving information in its definition). This fact might have decreased the need to account for heterogeneous residual variance in the RRM used for the genetic evaluations of the groups of structural problems and disease under the TL definition.

The higher residual variances estimated for FLa compared to TL and FLb indicate a worse model fit for this longevity definition compared to the others, which is likely related to the fact that uncertain information is inaccurately being assumed as certain under the FLa definition (i.e., the code 0 has been used for both situations, i.e., after the cow was culled or if the cow did not record a calf at the specific age). In this study, only one option of heterogenous residual variance was tested (i.e., 14 classes), which greatly increases the model’s complexity. One option to reduce the number of residual variance classes is to group similar classes together. Several methods can be used to group different classes of residual variance, such as self-organizing maps [50], change point [51], and visual inspection [52]. Testing different numbers of classes for the residual variance should be considered in subsequent studies in order to simplify the RRM.

### 4.3. Genetic Parameters

**Heritabilities.** Genetic analysis of longevity (or longevity-related traits, such as stayability and survival) are becoming popular in livestock breeding research [11,13,53]. However, to our best knowledge, there are no studies evaluating the impact of different culling reasons in the estimation of variance components and genetic parameters for longevity. In this study, different heritability estimates were obtained for each group of culling reason (Table 3 and Figure 3). For instance, higher heritability estimates were obtained for animals that died due to structural problems and disease, intermediate heritabilities were obtained for natural death, and lower heritabilities were obtained for performance, fertility, and miscellaneous. These results are due to the larger additive genetic variances observed for the groups of structural problems and disease (Figure S5, Supplementary Material), and the larger permanent environmental variances estimated for the groups of fertility, performance, and miscellaneous (Figure S6, Supplementary Material). Similar herd-year-season variances were estimated for all groups of culling reasons (Figure S7, Supplementary Material). Heritabilities estimated combining all groups of culling reasons were similar to the heritabilities estimated for the groups of performance, fertility, and miscellaneous. These findings indicate that combining all culling reasons for genetic and genomic evaluations without accounting for their genetic differences will likely weaken the genetic progress for longevity. Moreover, heritabilities estimated in this study suggest that direct genetic selection for longevity, regardless the group of culling reasons used, will result in improved longevity of North American Angus. Thus, combined with management strategies, including longevity in the breeding goals has the potential to reduce involuntary culling in the herds, which can minimize financial losses.

The main goal of using FL instead of TL in the genetic evaluation for longevity is to increase the probability that cows will not only be alive in the herd but also producing one calf per year, which will ensure an economic return for beef cattle producers [54,55,56]. In general, the average heritabilities estimated for FL_b_ tended to be higher than those estimated for FL_a_ and TL, indicating greater genetic progress is expected through direct selection for FL_b_. These results differ from those reported by Morales et al. [40], in which similar heritability estimates for length of true life (0.14) and length of productive life (0.14) were observed in the Retinta beef cattle breed. Even though there are similarities in the concepts used by the authors to define the traits and the TL and FLb definitions used in our study, the differences in the results are likely explained by the different statistical models used (Weibull proportional hazard vs. linear RRM).

Brzáková et al. [11] compared the use of two longevity definitions (i.e., probability of cow reappearance in the next parity, and the number of calvings at ages of 6.5, 7.5, 12.5, and 13.3 years) for the genetic evaluation of a beef cattle population in the Czech Republic, using single- and multiple-trait linear models. Despite the fact that the statistical models and the longevity definitions used in their study are not conceptually the same as in our study, the authors reported heritability estimates close to the ones found in our study when considering the group combining all culling reasons under the FLa definition (heritabilities ranging from 0.09 to 0.13). Similar heritabilities (ranging from 0.09 to 0.16) were also estimated for stayability (defined as probability that a cow had calved at least three times before 6.3 years) in Brazilian Nellore cattle [57]. On the other hand, higher heritability estimates (ranging from 0.18 to 0.25) were found for stayability (defined as stayability to calving, from 2 to 6 years) in Hereford cattle, using a traditional linear model [14]. Using an approach based on RRM similar to the one presented in our study, Jamrozik et al. [13] performed a genetic evaluation for stayability to consecutive calvings (defined similarly to the FLb definition used in our study) in Canadian Simmentals. The heritabilities estimated by the afore mentioned authors were, in general, similar to the heritabilities estimated in our study for the culling group of natural death under the FLb definition (heritabilities estimated by them ranged from 0.13 to 0. 35). Specifically for Aberdeen Angus, Roughsedge et al. [10] estimated a heritability of 0.13 for lifespan. Lifespan was defined by the authors to reflect the parity that was expected to be reached using average survival probabilities from parity to parity in the population [10]. For South African Angus cattle, heritabilities estimated using an animal threshold model ranged from 0.18 to 0.20 for stayability defined as the probability that a cow remained in the herd from 4 to 8 years of age [55].

In general, most peaks of heritability observed for the different longevity definitions analyzed in our study occurred between 4 to 7 years, which indicates that performing selection within this interval can help to accelerate the genetic gain for longevity. This is in agreement with Brzáková et al. [11], who reported that the highest heritabilities were estimated at 6.5 years. On the other hand, the highest heritability reported by Jamrozik et al. [13] was estimated at 2 years. For the majority of groups of culling reasons and longevity definitions analyzed in our study, smaller heritabilities were obtained at 2 and over 12 years compared to the intermediate age categories. These smaller heritabilities in the extremes are likely due to the lower genetic variability observed in these ages (Figure S5, Supplementary Material), which is a consequence of the quality control performed (i.e., all cows were required to have their first calving before 30 months of age), selection process, and reduced phenotypic variability (Figure S3, Supplementary Material). In this regard, the sudden increase in the heritability estimates observed at high ages specifically for the group of disease under the FLa definition (Figure 3b), and the groups of structural problems and performance under the FLb definition (Figure 3c) are likely due to a poor fit of a high order polynomial at these points when using heterogeneous residual variance. Moreover, various studies currently available in the literature have reported unstable heritability estimates in the extremes of the curve for several traits when using RRM based on high-order Legendre polynomials [50,58,59].

**Genetic correlations between ages.** Cattle longevity traits have been traditionally evaluated at 6 years-old, which leads to explicitly ignoring records from cows that are not yet 6 years-old or that are still alive beyond this age [17]. In our study, a comprehensive range of ages was evaluated using RRM (i.e., 2 to 15 years-old), which enabled us to use all the information available to identify the best time periods to perform the selection for increased longevity. In general, our findings showed that higher genetic correlations were observed at closer ages, and that the magnitude of the correlations decreased with increasing gap between ages (Figure 4). These findings are as expected with a RRM and in agreement with the ones reported by Jamrozik et al. [13], studying stayability to consecutive calvings using RRM in Canadian Simmental cattle.

The low average genetic correlations estimated when considering all ages (i.e., from 2 to 15 years; Table 4) is due to the negative genetic correlations found for ages greater than 12 years. These negative correlations are likely due to the reduced number of records in these age categories, which is a consequence of the selection process, combined to the instability of variance components in the extremes of the curve generated by poor fit of the high-order Legendre polynomials [50,58,59]. Regardless of this issue, Sánchez-Castro et al. [17] reported that the inclusion of older age records (e.g., 7 and 12 years) increases the EBV stability for stayability measured at the traditional 6 years old in Angus cows. In addition, the authors commented that accuracies (calculated according to the guidelines of the BIF [60]) obtained using RRM including the additional ages were higher than accuracies obtained with the RRM that only used data up to 6 years of age. Similarly, Bohmanova et al. [61] suggested that EBV accuracies increase when additional records were incorporated into the RRM. Thus, even though caution is advised when evaluating animals for longevity at ages greater than 12 years-old, inclusion of these records can be beneficial for the genetic and genomic evaluations of North American Angus cattle.

The genetic correlations estimated for ages between 3 and 12 years (Table 4) indicate that longevity up to 12 years-old can be well predicted using any time point within the mentioned interval. In this context, using EBVs predicted for early ages such as 4 or 5 years can help to shorten the generation interval for longevity, as well as accelerate genetic gain due to the higher heritabilities estimated at these ages (Figure 3). Similar findings were also reported in other studies. For instance, Jamrozik et al. [13] reported genetic correlations ranging from 0.74 to 0.99 between ages 3 to 8 years-old. Brzáková et al. [11] found that productive longevity measured at 7.5 years-old is a good indicator of longevity measured at 13.3 years-old (genetic correlations reported by the authors were above 0.84). In addition, Venot [62] reported high genetic correlations (0.95 for Charolais and 0.92 for Aubrac) for number of calvings estimated between 6.5 and 12.5 years-old, while evaluating length of productive life in beef cows.

Finding measures of longevity taken earlier in life is paramount, as true longevity is not known until the end of a cow’s life [11,17]. However, it is important to highlight that the genetic correlation estimated between 2 years and older ages was, in general, substantially lower than the genetic correlation estimated at 3 or 4 years and the other ages (Figure 4). These findings might be related to the fact that longevity at 2 years-old is likely more strongly associated with the analyzed culling reasons than older ages, as suggested by Cushman et al. [42] and Damiran et al. [32]. Therefore, it is advisable to avoid using EBVs predicted at 2 years-old to select animals when the goal is to improve longevity at older ages.

**Genetic correlations between culling reasons.** Even though culling reasons are currently being reported by the farmers for some dairy and beef cattle breeds, to our best knowledge, this information has not been used for genetic and genomic evaluations of longevity around the world [63,64]. The main reason for that is the complexity to account for multiple culling reasons in the statistical models [13]. Nonetheless, identifying the culling reasons allows the recognition of the proportion of voluntary and involuntary culling in the herd [9,15], which can influence management decisions. In addition, identifying the impact of the culling reasons in the estimation of variance components can contribute to obtaining more accurate EBVs and accelerate genetic progress for longevity.

In general, the genetic correlations estimated between the different culling groups were low (Table 5), indicating that longevity based on different culling reasons are genetically different traits. These low genetic correlations support the different heritabilities estimated for each culling reason (as previously discussed in the *Heritabilities* topic). Furthermore, these findings suggest that combining all culling reasons can have a negative impact in the selection program, as heritabilities estimated when considering all culling reasons together were low. However, genetically evaluating multiple longevity traits (e.g., one for each culling reason) can be challenging, as various trait EBVs would be generated. Thus, one strategy would be to develop a selection sub-index [65,66] for longevity, where different weights would be applied to each longevity indicator. In this context, greater weights can be used for more prevalent culling reasons (i.e., culling reasons with higher economic impact), such as fertility.

The highest average genetic correlations were found between the groups of animals culled due fertility and performance (Table 5), which might be a consequence of the strong relationship between these group of traits. For instance, a recent study performed by Pardo et al. [67] showed that there is a strong positive genetic correlation (0.98 ± 0.01) between fertility (i.e., age at first calving) and performance (i.e., pre-weaning average daily gain), in a beef cattle population composed by Angus, Hereford, and their crossbreeds raised in Argentina. In addition, positive and favorable genetic correlations were estimated between the number of calves at 4.4 years of age and weight gain calculated from weaning to yearling (0.42 ± 0.04) in Nellore cattle [68]. Speculations regarding the genetic correlations estimated between the other pair of culling reasons can be made, however, to a lesser extent. For instance, the genetic correlation estimated between the groups of structural problems and disease might be related to the impact of inadequate claw set and foot angle on the incidence of lameness [35]. However, in order to validate these findings and clarify the genetic relationship between longevity traits based on different culling reasons over time, single-step genome-wide association studies based on RRM [69,70] and multiple-trait analyses considering longevity and other recorded traits (such as heifer pregnancy, mature cow size, claw and foot angle) could be employed.

Longevity-related traits have been found to be genetically correlated to several other traits. For instance, Valente et al. [71] reported negative genetic correlations ranging from −0.03 ± 0.11 (between stayability and flight speed) to −0.24 ± 0.16 (between stayability and crush score) in Nellore cattle. Stronger genetic correlations were found by Martínez-Velázquez et al. [72] for scrotal circumference and stayability (0.76 ± 0.04) and between heifer fertility and stayability (0.57 ± 0.07) in a population composed by Charolais, Charbray, and Charolais–Zebu crosses. Costa et al. [57] reported genetic correlations between age at first calving and stayability ranging from −0.23 to −0.51, depending on the statistical model used for the analysis (linear-threshold, penalty-threshold, modified penalty-threshold, and linear-threshold-threshold model) in Nellore cattle.

**Genetic correlations between longevity definitions.** Even though the definitions used to describe longevity and longevity-related traits are still not clear in the literature, it seems that most studies in beef cattle have preferred the term stayability [12,13,14] to describe longevity. However, the definitions used to describe stayability are, sometimes, very similar to the definitions used in studies for other species, which have used the term longevity [9,48,73]. Similar definitions for longevity were also found in the literature under the terms survival [16,47] and productive life [30,40,62]. For simplicity, in our study the term longevity was used to describe both TL and FL. Thus, TL was compared to two definitions of FL: including or not missing records for cows without calving information at a specified age (FLb and FLa, respectively). The higher genetic correlations observed between TL and FL_b_ compared to between TL and FLa, and between FLa and FLb suggest that the information of death (represented by the code 0 in this study) has a higher impact than the information of calving (code 1) in the genetic evaluation of longevity. In addition, the average genetic correlations estimated between the different longevity definitions in our study indicate that the definition used in the breeding program can impact selection decisions (Table 6). The magnitude of the impact (i.e., proportion of sires commonly selected) is discussed in details in the “*Impact of longevity definition in the selection*” topic.

The average genetic correlations estimated between the different longevity definitions found in our study corroborate with the ones reported by Martinez et al. [14], evaluating stayability to six ages (from 1 to 6 years) and stayability to calving and weaning (both from 2nd to 6th) in Hereford cows. The mentioned authors reported moderate genetic correlations between stayability to six ages and the other definitions (from 0.51 to 0.57) and high genetic correlations between stayability to calving and stayability to weaning (0.86), suggesting possibly re-ranking of sires depending on the trait definition. On the other hand, Morales et al. [40] estimated high EBV correlations (above 0.96) for length of true life, length of productive life, and number of calvings in the Retinta breed. The authors recommended the use of number of calvings for subsequent genetic and genomic evaluations in the same population due to its higher heritability and easier data access compared to the other traits [40]. Brzáková et al. [11] compared the use of two longevity definitions (i.e., probability of cow reappearance in the next parity and the number of calvings at different ages) using a beef cattle population from Czech Republic. Even though the authors did not evaluate the genetic correlation between both longevity definitions, they concluded that evaluating the number of calvings (mainly at 7.5 and 13.3 years-old) is preferred to avoid cows that do not produce one calf per year. Similarly to our study, Brzáková et al. [11] commented that there was enough additive genetic variance for all traits analyzed.

Comparing the average genetic correlations estimated for the longevity definitions between the different culling reasons, lower genetic correlations tended to be observed for the culling group of fertility (Table 6). These lower correlations are likely related to the longevity definitions used, which differ mainly regarding the use of calving information. Furthermore, as expected, similar average genetic correlations were estimated for the other culling groups, considering either all ages (i.e., from 2 to 15 years), or ages within the interval of 3 and 12 years (Table 6).

### 4.4. Impact of Longevity Definition in the Selection Schemes

In order to facilitate the comparison of the impact of different longevity definitions in the breeding program, the sires’ EBVs were expressed in terms of EDL (only for sires with a minimum of 5 daughters with longevity data). Using EDL to better understand the response to selection has been routine in genetic and genomic evaluations of several functional traits in both beef and dairy cattle [29,63]. A higher proportion of commonly-selected sires was observed between TL and FLb (Table 7), which can be explained by the higher genetic correlation estimated between these longevity definitions (Table 6). Likewise, the average EDL predicted for TL and FLb were more similar than EDL predicted for TL and FLa, and FLa and FLb (Table 9, Table 10 and Table 11). However, larger standard deviations were found for the average EDL predicted using the FLb definition compared to the TL and FLa definitions, which is a consequence of the greater genetic variability found for this longevity definition for the majority of groups of culling reasons (Figure S5, Supplementary Material). The greater dispersion of EDL (as well as sires’ EBVs) using FLb is favorable for selection, as it can increase the genetic gain per time unit [74]. Moreover, our findings suggest that different sires would be selected based on each longevity definition (Table 7).

The average differences between the top and bottom sires calculated for all longevity definitions (Table 9, Table 10 and Table 11) suggest that daughters sired by the top 1% bulls are about twice as likely to remain longer in the herd than daughters sired by the bottom 1%. Greater average differences between the top and bottom sires tended to be found for the culling group of performance (Table 9, Table 10 and Table 11), which suggests that the ongoing selection for performance has impacted the longevity trait in North American Angus cattle. For instance, performance traits (e.g., accumulated productivity) have been found to be highly correlated (0.86 ± 0.03) with longevity-related traits in Nellore cattle [75]. Thus, selecting animals for improved genetic performance might have also contributed to increase the lifetime of Angus cows in the herd. However, it is important to highlight that multiple-trait analyses including longevity and performance data in Angus cattle are required to validate this theory. The smallest average differences found for the group of natural death compared to the other culling groups might indicate that no effective direct selection has been performed for longevity in North American Angus cattle (Table 9, Table 10 and Table 11). This finding might be related to the fact that the American Angus Association [33] and the Canadian Angus Association [34] currently do not perform genetic evaluations for longevity traits.

As expected, selecting animals at an older age increased the prediction accuracy (Table 8, and Figures S9 and S10 in the Supplementary Material), which is likely related to the higher genetic correlation found between adjacent ages (Table 4). However, the averages of improvement in prediction accuracy when compared to the previous age were the highest when selection was performed at 3 or 4 years of age. This suggests that selecting animals at 4 years-old might be more efficient (in terms of correlation of expected and observed values) in the long-term. However, as genetic parameters are usually population-specific, it is advised to test the optimal age to perform selection for longevity traits in other populations. The similar average prediction accuracies calculated for the daughters’ average culling age and proportion of daughters alive at 6 years suggest that the decision of using one or the other EDL would rely exclusively on the ultimate breeding goal. Nonetheless, in most cases, the FLb definition tended to yield slightly higher accuracies compared to TL and FLa, which indicates that the inclusion of FLb in the breeding programs might have advantages (e.g., higher genetic gain) compared to the others. Prediction accuracies estimated in our study ranged from low to moderate, which is a consequence of the heritabilities estimated in our study. These prediction accuracies can be further improved if adjusted for the accuracy of the EBV, or if genomic information are included in the analysis [76,77]. In this context, Ramos et al. [12] suggested that genomic information is always required to ensure high accuracies for longevity-related traits at early ages, because these traits are usually characterized as late-measured and sex-restricted traits.

## 5. Conclusions

This study was the first attempt to genetically evaluate longevity in North American Angus cattle based on large and comprehensive datasets. Random regression models considering heterogeneity of residual variance and fourth order Legendre orthogonal polynomials to describe the average, herd-year-season, additive genetic, and permanent environmental effects over ages were considered as optimal for most longevity definitions and culling reasons evaluated. Moreover, our findings indicate that the functional longevity definition considering missing records (FLb) is preferred for the genetic evaluation of longevity in North American Angus cattle due to its higher heritability estimates and prediction accuracies for the expected daughter performances. Our results also suggest that longevity based on different groups of culling reasons should not be analyzed together, as they are genetically different traits. Among the different time-periods assessed to perform selection, the age of 4 years is recommended in order to improve selection responses for increased longevity in North American Angus cattle.

## Figures and Tables

**Table 1:** Description of the different groups of culling reason in North American Angus cattle.

Group	Class	N	N_Total_
Natural Death	Died due to non-apparent reasons	55,232	150,229
	Culled due to age	94,997	
Structural Problems	Eye problem	499	24,804
	Body structure	13,101	
	Teat and udder conformation	5845	
	Rectal prolapse	73	
	Vaginal prolapse	103	
	Feet conformation	5183	
Disease	Illness or disease	4994	4994
Fertility	Fertility	124,696	154,419
	Missed calving opportunity	29,723	
Performance	Productivity or progeny performance	53,837	62,005
	Temperament	8168	
Miscellaneous	Culled as miscellaneous Sold as commercial	44,563	208,092
		163,529	
All Reasons	All	604,543	604,543

**Table 2:** Deviance information criterion (DIC) and the posterior model probabilities (PMP, inside parenthesis) calculated for each group of culling reason and longevity definition, using random regression models based on the fourth order Legendre orthogonal polynomials and assuming homogeneous or heterogeneous residual variance.

Culling Reason	Residual	DIC (PMP)		
		^1^TL	^2^FL_a_	^3^FL_b_
Natural death	Homogeneous	−14,333,660 (0)	−2,998,505 (0)	−35,724,631,037 (0)
	Heterogeneous	−97,527,370 (1)	−7,435,206 (1)	−162,024,103,613 (1)
Structural Problems	Homogeneous	−1,947,409 (1)	−937,946 (0)	−3,032,840,387 (0)
	Heterogeneous	−275,489 (0)	−2,624,539 (1)	−11,343,283,933 (1)
Disease	Homogeneous	−432,592 (1)	−219,798 (0)	−1,728,658,673 (0)
	Heterogeneous	−63,888 (0)	−765,673 (1)	−69,837,105,629 (1)
Fertility	Homogeneous	−15,460,202 (0)	−5,952,231 (0)	−15,053,722,233 (0)
	Heterogeneous	−137,123,460 (1)	−13,167,214 (1)	−691,563,543,599 (1)
Performance	Homogeneous	−5,518,605 (0)	−3,418,738 (0)	−6,421,240,367 (0)
	Heterogeneous	−52,327,651 (1)	−7,036,948 (1)	−28,107,833,193 (1)
Miscellaneous	Homogeneous	−19,534,605 (0)	−7,195,212 (0)	−26,940,276,644 (0)
	Heterogeneous	−153,303,945 (1)	−15,449,397 (1)	−26,942,298,626 (1)
All Reasons	Homogeneous	−32,816,459 (0)	−20,020,368 (0)	−85,088,486,564 (0)
	Heterogeneous	−44,115,210 (1)	−20,113,943 (1)	−93,934,683,621 (1)

^1^TL: Traditional longevity. ^2^FL_a_: Functional longevity assuming 0 after the cow was culled or if the cow did not record a calf at the specific age. ^3^FL_b_: Functional longevity assuming 0 only after the cow was culled, and missing records when no information of calving was found at the specific age.

**Table 3:** Average heritabilities (±SE) estimated considering all ages (i.e., from 2 to 15 years) and ages between 3 and 12 years-old, for all longevity definitions and culling reasons.

Ages	Culling Reason	Longevity Definition		
		^1^TL	^2^FL_a_	^3^FL_b_
All	Natural death	0.19 ± 0.02	0.15 ± 0.02	0.21 ± 0.02
	Structural problems	0.23 ± 0.04	0.17 ± 0.02	0.24 ± 0.03
	Disease	0.19 ± 0.03	0.19 ± 0.02	0.25 ± 0.02
	Fertility	0.07 ± 0.01	0.07 ± 0.01	0.08 ± 0.01
	Performance	0.10 ± 0.02	0.08 ± 0.01	0.10 ± 0.01
	Miscellaneous	0.08 ± 0.01	0.07 ± 0.01	0.08 ± 0.01
	All	0.11 ± 0.01	0.09 ± 0.01	0.13 ± 0.01
3 to 12 years	Natural death	0.22 ± 0.02	0.17 ± 0.02	0.24 ± 0.02
	Structural problems	0.30 ± 0.03	0.21 ± 0.02	0.28 ± 0.03
	Disease	0.24 ± 0.02	0.23 ± 0.02	0.28 ± 0.02
	Fertility	0.09 ± 0.01	0.09 ± 0.01	0.10 ± 0.01
	Performance	0.13 ± 0.02	0.10 ± 0.01	0.13 ± 0.01
	Miscellaneous	0.08 ± 0.00	0.09 ± 0.01	0.10 ± 0.01
	All	0.12 ± 0.00	0.09 ± 0.01	0.15 ± 0.01

^1^TL: Traditional longevity. ^2^FL_a_: Functional longevity assuming 0 after the cow was culled or if the cow did not record a calf at the specified age. ^3^FL_b_: Functional longevity assuming 0 only after the cow was culled, and missing records when no calving information was found at the specified age.

**Table 4:** Average (±SE) genetic correlations estimated considering all ages (i.e., from 2 to 15 years) and ages between 3 and 12 years-old for all longevity definitions and known culling reasons.

Ages	Culling Reason	Longevity Definition			Average
		^1^TL	^2^FL_a_	^3^FL_b_	
All	Natural death	0.45 ± 0.12	0.56 ± 0.11	0.46 ± 0.12	0.49
	Structural problems	0.38 ± 0.13	0.36 ± 0.14	0.37 ± 0.14	0.37
	Disease	0.36 ± 0.12	0.35 ± 0.13	0.37 ± 0.13	0.36
	Fertility	0.32 ± 0.13	0.40 ± 0.13	0.32 ± 0.13	0.35
	Performance	0.43 ± 0.11	0.48 ± 0.12	0.44 ± 0.12	0.45
Average		0.39	0.43	0.39	0.40
3 to 12 years	Natural death	0.74 ± 0.06	0.84 ± 0.05	0.74 ± 0.06	0.77
	Structural problems	0.75 ± 0.11	0.76 ± 0.12	0.76 ± 0.13	0.76
	Disease	0.71 ± 0.11	0.68 ± 0.12	0.71 ± 0.12	0.70
	Fertility	0.65 ± 0.10	0.72 ± 0.12	0.64 ± 0.12	0.67
	Performance	0.74 ± 0.10	0.81 ± 0.09	0.75 ± 0.10	0.77
Average		0.72	0.76	0.72	0.73

^1^TL: Traditional longevity. ^2^FL_a_: Functional longevity assuming 0 after the cow was culled or if the cow did not record a calf at the specified age. ^3^FL_b_: Functional longevity assuming 0 only after the cow was culled, and missing records when no information of calving was found at the specific age.

**Table 5:** Average (±SE) genetic correlations estimated between the different culling reasons considering all ages (i.e., from 2 to 15 years) and ages between 3 and 12 years-old, for all longevity definitions.

Ages	Culling Reason		Longevity Definition			Average
	1	2	^1^TL	^2^FL_a_	^3^FL_b_	
All	Natural death	Structural problems	0.13 ± 0.03	0.17 ± 0.05	0.10 ± 0.04	0.13
		Disease	−0.02 ± 0.02	0.08 ± 0.03	−0.01 ± 0.03	0.02
		Fertility	0.16 ± 0.05	0.19 ± 0.05	0.06 ± 0.05	0.14
		Performance	0.20 ± 0.04	0.18 ± 0.05	0.15 ± 0.06	0.18
	Structural problems	Disease	0.10 ± 0.04	0.15 ± 0.06	0.11 ± 0.06	0.12
		Fertility	0.11 ± 0.03	0.21 ± 0.05	0.13 ± 0.06	0.15
		Performance	0.20 ± 0.05	0.17 ± 0.05	0.11 ± 0.03	0.16
	Disease	Fertility	−0.02 ± 0.05	0.10 ± 0.04	0.07 ± 0.05	0.05
		Performance	−0.01 ± 0.04	0.04 ± 0.04	−0.02 ± 0.02	0.00
	Fertility	Performance	0.30 ± 0.06	0.23 ± 0.05	0.20 ± 0.05	0.24
Average						0.12
3 to 12 years	Natural death	Structural problems	0.19 ± 0.02	0.27 ± 0.03	0.20 ± 0.04	0.22
		Disease	−0.05 ± 0.02	0.09 ± 0.03	−0.03 ± 0.03	0.00
		Fertility	0.26 ± 0.05	0.25 ± 0.04	0.09 ± 0.05	0.20
		Performance	0.28 ± 0.02	0.27 ± 0.04	0.19 ± 0.07	0.25
	Structural problems	Disease	0.20 ± 0.03	0.30 ± 0.05	0.26 ± 0.04	0.25
		Fertility	0.17 ± 0.02	0.38 ± 0.02	0.26 ± 0.05	0.27
		Performance	0.32 ± 0.02	0.28 ± 0.02	0.17 ± 0.02	0.26
	Disease	Fertility	0.10 ± 0.05	0.19 ± 0.04	0.20 ± 0.03	0.16
		Performance	−0.01 ± 0.03	0.05 ± 0.05	−0.01 ± 0.02	0.01
	Fertility	Performance	0.46 ± 0.03	0.36 ± 0.03	0.35 ± 0.03	0.39
Average						0.20

^1^TL: Traditional longevity. ^2^FL_a_: Functional longevity assuming 0 after the cow was culled or if the cow did not record a calf at the specified age. ^3^FL_b_: Functional longevity assuming 0 only after the cow was culled, and missing records when no information of calving was found at the specified age.

**Table 6:** Average (±SE) genetic correlations estimated between the different longevity definitions considering all ages (i.e., from 2 to 15 years) and ages between 3 and 12 years-old, for all groups of known culling reason.

Ages	Culling Reason	^1^Longevity Definitions		
		TL vs. FLa	TL vs. FLb	FLa vs. FLb
All	Natural death	0.39 ± 0.08	0.48 ± 0.12	0.45 ± 0.08
	Structural problems	0.38 ± 0.12	0.41 ± 0.13	0.39 ± 0.13
	Disease	0.35 ± 0.11	0.39 ± 0.13	0.37 ± 0.11
	Fertility	0.32 ± 0.08	0.36 ± 0.13	0.34 ± 0.08
	Performance	0.46 ± 0.10	0.47 ± 0.11	0.51 ± 0.11
Average		0.38	0.42	0.41
3 to 12 years	Natural death	0.58 ± 0.04	0.75 ± 0.06	0.6 ± 0.04
	Structural problems	0.72 ± 0.09	0.76 ± 0.10	0.72 ± 0.10
	Disease	0.63 ± 0.09	0.72 ± 0.09	0.63 ± 0.09
	Fertility	0.52 ± 0.06	0.67 ± 0.09	0.57 ± 0.07
	Performance	0.73 ± 0.07	0.76 ± 0.08	0.74 ± 0.07
Average		0.64	0.73	0.65

^1^Longevity definitions are: traditional longevity (TL); functional longevity assuming 0 after the cow was culled or if the cow did not record a calf at the specified age (FL_a_); and functional longevity assuming 0 only after the cow was culled, and missing records when no information of calving was found at the specified age (FL_b_).

**Table 7:** Average (± SE) proportion of animals commonly selected between the different ^1^longevity definitions considering all ages (i.e., from 2 to 15 years) and ages between 3 and 12 years-old, for the top 1% and 10% sires, with more than 5 daughters with phenotypic records, of all groups of known culling reason.

Ages	Culling Reason	Top 1%			Top 10%		
		TL vs. FLa	TL vs. FLb	FLa vs. FLb	TL vs. FLa	TL vs. FLb	FLa vs. FLb
All	Natural death	44.29 ± 3.20	87.56 ± 3.22	47.56 ± 2.66	57.46 ± 1.58	90.19 ± 1.87	59.99 ± 1.49
	Structural problems	64.15 ± 3.82	86.81 ± 3.78	65.60 ± 3.80	75.00 ± 2.41	90.01 ± 2.00	76.19 ± 2.47
	Disease	56.67 ± 5.49	87.84 ± 2.89	57.97 ± 5.29	69.23 ± 3.59	89.79 ± 2.29	71.04 ± 3.12
	Fertility	36.47 ± 3.92	78.66 ± 5.42	40.77 ± 2.45	52.60 ± 3.12	83.50 ± 2.99	55.96 ± 1.86
	Performance	69.99 ± 5.90	82.55 ± 5.93	71.84 ± 5.82	76.15 ± 3.36	86.78 ± 3.21	77.78 ± 3.05
Average		54.31	84.68	56.75	66.09	88.05	68.19
3 to 12 years	Natural death	45.32 ± 4.02	91.49 ± 0.89	48.74 ± 3.68	58.37 ± 1.88	92.46 ± 0.69	60.34 ± 1.52
	Structural problems	66.73 ± 4.69	92.50 ± 1.02	67.31 ± 4.61	76.30 ± 3.05	93.24 ± 0.70	76.65 ± 3.04
	Disease	63.56 ± 5.48	93.51 ± 0.77	64.07 ± 5.39	73.33 ± 3.70	93.89 ± 0.31	73.62 ± 3.63
	Fertility	43.65 ± 2.07	88.52 ± 1.45	44.62 ± 1.86	58.65 ± 1.29	89.77 ± 0.97	59.41 ± 1.15
	Performance	79.12 ± 5.26	92.67 ± 0.85	80.30 ± 4.72	81.47 ± 2.86	92.71 ± 0.44	82.22 ± 2.67
Average		59.68	91.74	61.01	69.62	92.41	70.45

^1^Longevity definitions are: traditional longevity (TL); functional longevity assuming 0 after the cow was culled or if the cow did not record a calf at the specified age (FL_a_); and functional longevity assuming 0 only after the cow was culled, and missing records when no information of calving was found at the specified age (FL_b_).

**Table 8:** Average (±SE) prediction accuracy of the expected daughter longevity (EDL) using different ages at selection (from 2 to 6 years-old), for all longevity definitions.

^1^EDL	Age at Selection	^2^Longevity Definition			Average	^3^Average Improvement (%)
		TL	FLa	FLb		
Average Culling Age	2	0.09 ± 0.03	0.14 ± 0.01	0.11 ± 0.02	0.11	-
	3	0.09 ± 0.02	0.14 ± 0.01	0.23 ± 0.02	0.15	35.29
	4	0.26 ± 0.02	0.24 ± 0.02	0.27 ± 0.02	0.26	67.39
	5	0.29 ± 0.02	0.29 ± 0.02	0.29 ± 0.02	0.29	12.99
	6	0.31 ± 0.02	0.27 ± 0.02	0.31 ± 0.02	0.30	2.30
Average		0.21	0.22	0.24		
Proportion Alive at 6 Years	2	0.08 ± 0.02	0.14 ± 0.03	0.11 ± 0.03	0.11	-
	3	0.23 ± 0.03	0.22 ± 0.01	0.25 ± 0.03	0.23	112.12
	4	0.28 ± 0.03	0.24 ± 0.02	0.28 ± 0.03	0.27	14.29
	5	0.30 ± 0.03	0.30 ± 0.03	0.31 ± 0.03	0.30	12.50
	6	0.31 ± 0.03	0.27 ± 0.03	0.31 ± 0.03	0.30	0.00
Average		0.24	0.23	0.25		
Proportion Alive at 9 Years	2	0.03 ± 0.01	0.07 ± 0.02	0.07 ± 0.02	0.06	-
	3	0.10 ± 0.01	0.14 ± 0.04	0.13 ± 0.01	0.12	105.88
	4	0.16 ± 0.02	0.18 ± 0.03	0.16 ± 0.02	0.17	42.86
	5	0.22 ± 0.02	0.22 ± 0.02	0.21 ± 0.02	0.22	30.00
	6	0.26 ± 0.02	0.24 ± 0.02	0.26 ± 0.02	0.25	16.92
Average		0.15	0.17	0.18		
Proportion Alive at 12 Years	2	0.03 ± 0.01	0.07 ± 0.01	0.07 ± 0.01	0.06	-
	3	0.04 ± 0.02	0.07 ± 0.03	0.06 ± 0.02	0.06	0.00
	4	0.06 ± 0.02	0.08 ± 0.03	0.07 ± 0.02	0.07	31.25
	5	0.09 ± 0.03	0.09 ± 0.03	0.09 ± 0.03	0.09	28.57
	6	0.11 ± 0.03	0.11 ± 0.03	0.11 ± 0.03	0.11	22.22
Average		0.07	0.09	0.08		

^1^EDL was calculated for the daughter’s average culling age and the proportion of daughters alive at 6, 9, and 12 years-old. ^2^Longevity definitions are: traditional longevity (TL); functional longevity assuming 0 after the cow was culled or if the cow did not record a calf at the specified age (FL_a_); and functional longevity assuming 0 only after the cow was culled, and missing records when no information of calving was found at the specified age (FL_b_). ^3^Average improvement was calculated based on the average of the previous age, in percentage.

**Table 9:** Average (±SE) expected daughter longevity (EDL) predicted for the top and bottom 1% and 10% sires, considering different groups of known culling reasons and selection at 4 years-old for traditional longevity.

Culling Reason	^1^EDL	1%			10%		
		Top	Bottom	^2^Dif (%)	Top	Bottom	^2^Dif (%)
Natural Death	Culling age	11.57 ± 0.2	7.92 ± 0.06	31.55	10.72 ± 0.03	8.6 ± 0.03	19.78
	6 years	0.99 ± 0.03	0.56 ± 0.01	43.43	0.93 ± 0.00	0.66 ± 0.01	29.03
	9 years	0.82 ± 0.02	0.42 ± 0.01	48.78	0.70 ± 0.00	0.51 ± 0.00	27.14
	12 years	0.29 ± 0.01	0.15 ± 0.00	48.28	0.27 ± 0.00	0.19 ± 0.00	29.63
Average				43.01			26.40
Structural Problems	Culling age	9.42 ± 0.03	6.01 ± 0.05	36.20	8.78 ± 0.02	6.61 ± 0.02	24.72
	6 years	0.95 ± 0.01	0.3 ± 0.01	68.42	0.81 ± 0.00	0.43 ± 0.00	46.91
	9 years	0.46 ± 0.00	0.12 ± 0.00	73.91	0.40 ± 0.00	0.18 ± 0.00	55.00
	12 years	0.06 ± 0.00	0.03 ± 0.00	50.00	0.05 ± 0.00	0.03 ± 0.00	40.00
Average				57.13			41.66
Disease	Culling age	8.99 ± 0.03	5.05 ± 0.08	43.83	8.41 ± 0.03	5.95 ± 0.06	29.25
	6 years	0.85 ± 0.00	0.14 ± 0.01	83.53	0.74 ± 0.01	0.32 ± 0.01	56.76
	9 years	0.41 ± 0.00	0.08 ± 0.01	80.49	0.35 ± 0.00	0.15 ± 0.00	57.14
	12 years	0.06 ± 0.00	0.04 ± 0.00	33.33	0.06 ± 0.00	0.05 ± 0.00	16.67
Average				60.29			39.95
Fertility	Culling age	7.78 ± 0.05	5.18 ± 0.03	33.42	7.30 ± 0.02	5.61 ± 0.02	23.15
	6 years	0.61 ± 0.01	0.25 ± 0.01	59.02	0.56 ± 0.01	0.32 ± 0.00	42.86
	9 years	0.28 ± 0.00	0.12 ± 0.00	57.14	0.25 ± 0.00	0.15 ± 0.00	40.00
	12 years	0.04 ± 0.00	0.03 ± 0.00	25.00	0.04 ± 0.00	0.03 ± 0.00	25.00
Average				43.64			32.75
Performance	Culling age	8.75 ± 0.05	4.91 ± 0.05	43.89	8.11 ± 0.02	5.61 ± 0.02	30.83
	6 years	0.75 ± 0.01	0.23 ± 0.01	69.33	0.67 ± 0.00	0.33 ± 0.00	50.75
	9 years	0.40 ± 0.01	0.08 ± 0.01	80.00	0.33 ± 0.00	0.13 ± 0.00	60.61
	12 years	0.11 ± 0.00	0.01 ± 0.00	90.91	0.09 ± 0.00	0.01 ± 0.00	88.89
Average				71.03			57.77

^1^EDL was calculated for the daughter’s average culling age and the proportion of daughters alive at 6, 9, and 12 years-old. ^2^Dif (%) is the average difference between top and bottom sires.

**Table 10:** Average (±SE) expected daughter longevity (EDL) predicted for the top and bottom 1% and 10% sires, considering different groups of known culling reasons and selection at 4 years-old for functional longevity assuming 0 after the cow was culled or if the cow did not record a calf at the specified age.

Culling Reason	^1^EDL	1%			10%		
		Top	Bottom	^2^Dif (%)	Top	Bottom	^2^Dif (%)
Natural Death	Culling age	11.26 ± 0.05	7.79 ± 0.04	30.82	10.75 ± 0.02	8.48 ± 0.03	21.12
	6 years	0.98 ± 0.01	0.57 ± 0.01	41.84	0.92 ± 0.01	0.65 ± 0.01	29.35
	9 years	0.83 ± 0.01	0.35 ± 0.01	57.83	0.76 ± 0.00	0.44 ± 0.00	42.11
	12 years	0.30 ± 0.00	0.13 ± 0.00	56.67	0.28 ± 0.00	0.17 ± 0.00	39.29
Average				46.79			32.96
Structural Problems	Culling age	9.19 ± 0.02	6.26 ± 0.04	31.88	8.76 ± 0.02	6.73 ± 0.02	23.17
	6 years	0.88 ± 0.00	0.38 ± 0.01	56.82	0.80 ± 0.00	0.46 ± 0.00	42.50
	9 years	0.45 ± 0.00	0.13 ± 0.01	71.11	0.41 ± 0.00	0.18 ± 0.00	56.10
	12 years	0.07 ± 0.00	0.02 ± 0.00	71.43	0.06 ± 0.00	0.03 ± 0.00	50.00
Average				57.81			42.94
Disease	Culling age	8.59 ± 0.02	5.79 ± 0.07	32.60	8.18 ± 0.03	6.43 ± 0.04	21.39
	6 years	0.78 ± 0.00	0.27 ± 0.01	65.38	0.71 ± 0.01	0.39 ± 0.01	45.07
	9 years	0.34 ± 0.01	0.17 ± 0.00	50.00	0.32 ± 0.00	0.21 ± 0.00	34.38
	12 years	0.07 ± 0.00	0.04 ± 0.00	42.86	0.06 ± 0.00	0.04 ± 0.00	33.33
Average				47.71			33.54
Fertility	Culling age	7.5 ± 0.03	5.29 ± 0.04	29.47	7.17 ± 0.01	5.74 ± 0.02	19.94
	6 years	0.62 ± 0.01	0.24 ± 0.01	61.29	0.56 ± 0.00	0.31 ± 0.00	44.64
	9 years	0.30 ± 0.00	0.09 ± 0.00	70.00	0.27 ± 0.00	0.13 ± 0.00	51.85
	12 years	0.04 ± 0.00	0.03 ± 0.00	25.00	0.04 ± 0.00	0.03 ± 0.00	25.00
Average				46.44			35.36
Performance	Culling age	8.62 ± 0.07	4.92 ± 0.07	42.92	8.05 ± 0.02	5.71 ± 0.03	29.07
	6 years	0.69 ± 0.01	0.28 ± 0.01	59.42	0.63 ± 0.00	0.37 ± 0.00	41.27
	9 years	0.41 ± 0.01	0.22 ± 0.01	46.34	0.35 ± 0.00	0.29 ± 0.00	17.15
	12 years	0.13 ± 0.00	0.04 ± 0.00	69.23	0.10 ± 0.00	0.06 ± 0.00	40.00
Average				54.48			31.87

^1^EDL was calculated for the daughter’s average culling age and the proportion of daughters alive at 6, 9, and 12 years-old. ^2^Dif (%) is the average difference between top and bottom sires.

**Table 11:** Average (±SE) expected daughter longevity (EDL) predicted for the top and bottom 1% and 10% sires, considering different groups of known culling reasons and selection at 4 years-old for functional longevity assuming 0 after the cow was culled, and missing records when no information of calving was found at the specified age.

Culling Reason	^1^EDL	1%			10%		
		Top	Bottom	^2^Dif (%)	Top	Bottom	^2^Dif (%)
Natural Death	Culling age	11.56 ± 0.22	7.91 ± 0.06	31.57	10.74 ± 0.04	8.58 ± 0.03	20.11
	6 years	0.99 ± 0.03	0.57 ± 0.01	42.19	0.93 ± 0.00	0.65 ± 0.00	30.11
	9 years	0.81 ± 0.02	0.42 ± 0.01	48.15	0.72 ± 0.00	0.49 ± 0.00	31.94
	12 years	0.29 ± 0.01	0.16 ± 0.00	44.83	0.26 ± 0.00	0.19 ± 0.00	26.92
Average				41.69			27.27
Structural Problems	Culling age	9.43 ± 0.03	6.00 ± 0.05	36.37	8.79 ± 0.02	6.60 ± 0.02	24.91
	6 years	0.95 ± 0.01	0.30 ± 0.01	68.42	0.83 ± 0.00	0.42 ± 0.00	49.40
	9 years	0.46 ± 0.00	0.12 ± 0.01	73.91	0.40 ± 0.00	0.18 ± 0.00	55.00
	12 years	0.06 ± 0.00	0.03 ± 0.00	50.00	0.05 ± 0.00	0.03 ± 0.00	40.00
Average				57.18			42.33
Disease	Culling age	8.98 ± 0.03	5.05 ± 0.09	43.76	8.41 ± 0.03	5.96 ± 0.06	29.13
	6 years	0.85 ± 0.00	0.14 ± 0.02	83.53	0.75 ± 0.01	0.31 ± 0.01	58.67
	9 years	0.40 ± 0.00	0.08 ± 0.01	80.00	0.35 ± 0.00	0.15 ± 0.01	57.14
	12 years	0.06 ± 0.01	0.04 ± 0.00	33.33	0.06 ± 0.00	0.05 ± 0.00	16.67
Average				60.16			40.40
Fertility	Culling age	7.78 ± 0.06	5.17 ± 0.03	33.55	7.31 ± 0.02	5.61 ± 0.02	23.26
	6 years	0.62 ± 0.01	0.26 ± 0.01	58.06	0.56 ± 0.00	0.32 ± 0.00	42.86
	9 years	0.28 ± 0.00	0.12 ± 0.00	57.14	0.25 ± 0.00	0.15 ± 0.00	40.00
	12 years	0.05 ± 0.00	0.02 ± 0.00	60.00	0.04 ± 0.00	0.03 ± 0.00	25.00
Average				52.19			32.78
Performance	Culling age	8.76 ± 0.07	4.90 ± 0.07	44.06	8.15 ± 0.02	5.61 ± 0.02	31.17
	6 years	0.75 ± 0.01	0.23 ± 0.01	69.33	0.67 ± 0.01	0.33 ± 0.00	50.75
	9 years	0.37 ± 0.01	0.06 ± 0.01	83.78	0.32 ± 0.00	0.12 ± 0.00	62.50
	12 years	0.11 ± 0.00	0.02 ± 0.00	81.82	0.09 ± 0.00	0.04 ± 0.00	55.56
Average				69.75			49.99

^1^EDL was calculated for the daughter’s average culling age and the proportion of daughters alive at 6, 9, and 12 years-old. ^2^Dif (%) is the average difference between top and bottom sires.

## Supplementary Materials

The following are available online at https://www.mdpi.com/2076-2615/10/12/2410/s1. Table S1: Deviance information criterion and the posterior model probabilities calculated for each group of culling reasons and longevity definitions, using random regression models based on different polynomial orders and homogeneous residual variance. Table S2: Average difference between expected daughter performances from the top and bottom 1% and 10% sires, calculated for all ages at selection (i.e., from 2 to 6 years), considering all groups of culling reasons and the traditional longevity definition. Table S3: Average difference between expected daughter performances from the top and bottom 1% and 10% sires, calculated for all ages at selection (i.e., from 2 to 6 years), considering all groups of culling reasons and the functional longevity definition assuming 0 after the cow was culled or if the cow did not record a calf at the specified age. Table S4: Average difference between expected daughter performances from the top and bottom 1% and 10% sires, calculated for all ages at selection (i.e., from 2 to 6 years), considering all groups of culling reasons and the functional longevity definition assuming 0 after the cow was culled, and missing records when no information of calving was found at the specified age. Figure S1: Number of cows by culling age (cumulative) inside each class of culling reasons. Figure S2: Number of cows by number of calvings inside each class of culling reasons. Figure S3: Proportion of codes 1, 0, and missing observed for each longevity definition over the ages. Figure S4: Heritabilities estimated over the different ages, for all groups of culling reasons and longevity definitions, using homogeneous residual variance. Figure S5: Additive genetic variance estimated over the different ages, for all groups of culling reasons and longevity definitions, using heterogeneous residual variance. Figure S6: Permanent environmental variance estimated over the different ages, for all groups of culling reasons and longevity definitions, using heterogeneous residual variance. Figure S7: Herd-year-season variance estimated over the different ages, for all groups of culling reasons and longevity definitions, using heterogeneous residual variance. Figure S8: Genetic correlations estimated between the different longevity definitions over ages, for all groups of known culling reasons. Figure S9: Prediction accuracy of the daughter’s average culling age calculated inside each group of known culling reasons and longevity definitions. Figure S10: Prediction accuracy of the expected daughter longevity (EDL) calculated inside each group of known culling reasons and longevity definitions.
