# Supplementary material for: Using Random Regression Models to Genetically Evaluate Functional Longevity Traits in North American Angus Cattle
Source: Animals (Basel). 2020 Dec 16;10(12):2410. doi: 10.3390/ani10122410 (PMC7766511; doi:10.3390/ani10122410)
Supplement: Supplementary file 1 [file animals-10-02410-s001.zip › SupplementaryMaterial_Final.docx]

**SUPPLEMENTARY MATERIAL**

**Table S1.** Deviance information criterion (DIC) and the posterior model probabilities (PMP, inside parenthesis) calculated for each group of culling reasons and longevity definition, using random regression models based on different polynomial orders and homogeneous residual variance.

| **Culling reason** | **^1^Model** | **DIC (PMP)** | | |
| --- | --- | --- | --- | --- |
|  |  | **^2^TL** | **^3^FL_a_** | **^4^FL_b_** |
| Natural death | LEG2 | -9,102,452 (0) | -679,755 (0) | -35,723,170,847 (0) |
|  | LEG3 | -12,062,647 (0) | -1,966,094 (0) | -35,723,841,309 (0) |
|  | LEG4 | -14,333,660 (1) | -2,998,505 (1) | -35,724,631,037 (1) |
| Structural problems | LEG2 | -1,033,907 (0) | -423,424 (0) | -3,032,346,371 (0) |
|  | LEG3 | -1,565,471 (0) | -711,235 (0) | -3,032,190,462 (0) |
|  | LEG4 | -1,947,409 (1) | -937,946 (1) | -3,032,840,387 (1) |
| Disease | LEG2 | -234,806 (0) | -113,867 (0) | -1,728,229,257 (0) |
|  | LEG3 | -350,834 (0) | -170,782 (0) | -1,728,292,303 (0) |
|  | LEG4 | -432,592 (1) | -219,798 (1) | -1,728,658,673 (1) |
| Fertility | LEG2 | -9,268,678 (0) | -3,223,640 (0) | -15,051,444,434 (0) |
|  | LEG3 | -12,816,906 (0) | -4,723,179 (0) | -15,050,671,536 (0) |
|  | LEG4 | -15,460,202 (1) | -5,952,231 (1) | -15,053,722,233 (1) |
| Performance | LEG2 | -3,086,954 (0) | -1,910,181 (0) | -6,420,562,365 (0) |
|  | LEG3 | -4,502,934 (0) | -2,714,666 (0) | -6,419,923,539 (0) |
|  | LEG4 | -5,518,605 (1) | -3,418,738 (1) | -6,421,240,367 (1) |
| Miscellaneous | LEG2 | -11,407,518 (0) | -3,891,685 (0) | -26,935,740,690 (0) |
|  | LEG3 | -16,095,245 (0) | -5,723,576 (0) | -26,934,750,130 (0) |
|  | LEG4 | -19,534,605 (1) | -7,195,212 (1) | -26,940,276,644 (1) |
| All reasons | LEG2 | -20,020,404 (0) | -9,491,570 (0) | -85,063,635,958 (0) |
|  | LEG3 | -28,423,144 (0) | -15,273,621 (0) | -85,063,557,515 (0) |
|  | LEG4 | -32,816,459 (1) | -20,020,368 (1) | -85,088,486,564 (1) |

^1^Model: LEG2, LEG3, and LEG4 were used to describe models based on second, third and fourth order Legendre orthogonal polynomials, respectively. ^2^TL: Traditional longevity. ^3^FL_a_: Functional longevity assuming 0 after the cow was culled or if the cow did not record a calf at the specific age. ^4^FL_b_: Functional longevity assuming 0 only after the cow was culled, and missing records when no information of calving was found at the specific age.

**Table S2.** Average difference (in percentage, %) between expected daughter longevity (EDL) from the top and bottom 1% and 10% sires, calculated for all ages at selection (i.e., from 2 to 6 years), considering all groups of culling reasons and the traditional longevity definition.

| **Culling reason** | **^1^EDL** | **1%** | | | | |  | **10%** | | | | |
| --- | --- | --- | --- | --- | --- | --- | --- | --- | --- | --- | --- | --- |
|  |  | **2 years** | **3 years** | **4 years** | **5 years** | **6 years** |  | **2 years** | **3 years** | **4 years** | **5 years** | **6 years** |
| Natural death | Culling age | 13.40 | 12.40 | 31.55 | 34.88 | 36.66 |  | 6.67 | 5.79 | 19.78 | 23.70 | 25.70 |
|  | 6 years | 14.94 | 37.00 | 43.43 | 48.08 | 49.04 |  | 7.23 | 21.35 | 29.03 | 33.68 | 35.42 |
|  | 9 years | 16.25 | 30.14 | 48.78 | 57.65 | 63.64 |  | 3.23 | 16.42 | 27.14 | 40.79 | 46.84 |
|  | 12 years | 16.00 | 35.71 | 48.28 | 50.00 | 58.06 |  | 8.33 | 20.00 | 29.63 | 37.04 | 42.86 |
| Average |  | 15.73 | 34.28 | 43.01 | 51.91 | 56.91 |  | 6.26 | 19.26 | 26.40 | 37.17 | 41.70 |
| Structural problems | Culling age | 6.23 | 12.92 | 36.20 | 38.32 | 39.23 |  | 4.07 | 5.19 | 24.72 | 26.61 | 27.85 |
|  | 6 years | 15.28 | 48.89 | 68.42 | 68.75 | 67.71 |  | 8.57 | 43.21 | 46.91 | 51.19 | 51.19 |
|  | 9 years | 14.29 | 56.10 | 73.91 | 82.00 | 86.79 |  | 10.71 | 40.54 | 55.00 | 61.90 | 68.18 |
|  | 12 years | 85.71 | 60.00 | 50.00 | 61.43 | 62.01 |  | 50.04 | 40.10 | 40.00 | 50.03 | 56.67 |
| Average |  | 38.43 | 55.00 | 57.13 | 70.73 | 72.17 |  | 23.10 | 41.25 | 41.66 | 54.37 | 58.68 |
| Disease | Culling age | 12.18 | 27.75 | 43.83 | 47.12 | 49.89 |  | 6.78 | 10.71 | 29.25 | 32.00 | 34.34 |
|  | 6 years | 55.56 | 75.00 | 83.53 | 86.90 | 87.21 |  | 18.18 | 53.42 | 56.76 | 61.33 | 63.16 |
|  | 9 years | 45.45 | 58.33 | 80.49 | 87.67 | 88.51 |  | 25.00 | 37.50 | 57.14 | 71.05 | 72.50 |
|  | 12 years | 75.00 | 20.00 | 33.33 | 47.14 | 57.50 |  | 60.00 | 17.89 | 16.67 | 42.86 | 47.14 |
| Average |  | 58.67 | 51.11 | 60.29 | 73.91 | 77.74 |  | 34.39 | 36.27 | 39.95 | 58.41 | 60.93 |
| Fertility | Culling age | 27.53 | 23.00 | 33.42 | 36.43 | 39.14 |  | 18.32 | 15.94 | 23.15 | 24.73 | 26.12 |
|  | 6 years | 38.18 | 51.67 | 59.02 | 64.06 | 68.75 |  | 25.49 | 38.18 | 42.86 | 46.43 | 49.12 |
|  | 9 years | 18.18 | 33.33 | 57.14 | 58.13 | 67.14 |  | 9.52 | 26.09 | 40.00 | 55.56 | 62.41 |
|  | 12 years | 25.00 | 25.00 | 25.00 | 60.00 | 78.00 |  | 20.00 | 24.02 | 25.00 | 46.05 | 49.90 |
| Average |  | 27.12 | 36.67 | 43.64 | 60.73 | 71.30 |  | 18.34 | 29.43 | 32.75 | 49.34 | 53.81 |
| Performance | Culling age | 23.34 | 20.67 | 43.89 | 47.51 | 49.19 |  | 14.85 | 13.03 | 30.83 | 33.49 | 34.80 |
|  | 6 years | 30.36 | 61.43 | 69.33 | 71.43 | 70.89 |  | 18.52 | 44.44 | 50.75 | 52.94 | 52.94 |
|  | 9 years | 34.62 | 65.63 | 80.00 | 85.24 | 95.80 |  | 24.00 | 48.28 | 60.61 | 75.00 | 81.58 |
|  | 12 years | 50.00 | 80.00 | 90.91 | 96.70 | 99.10 |  | 20.01 | 71.43 | 88.89 | 91.13 | 90.09 |
| Average |  | 38.32 | 69.02 | 71.03 | 84.46 | 88.60 |  | 20.84 | 54.72 | 57.77 | 73.02 | 74.87 |
| Overall |  | 31.83 | 43.24 | 55.02 | 62.85 | 67.24 |  | 18.50 | 30.98 | 39.71 | 49.19 | 52.35 |

^1^EDL was calculated for the daughter’s average culling age and the proportion of daughters alive at 6, 9, and 12 years-old.

**Table S3.** Average difference (in percentage, %) between expected daughter longevity (EDL) from the top and bottom 1% and 10% sires, calculated for all ages at selection (i.e., from 2 to 6 years), considering all groups of culling reasons and the functional longevity definition assuming 0 after the cow was culled or if the cow did not record a calf at the specified age.

| **Culling reason** | **^1^EDL** | **1%** | | | | |  | **10%** | | | | |
| --- | --- | --- | --- | --- | --- | --- | --- | --- | --- | --- | --- | --- |
|  |  | **2 years** | **3 years** | **4 years** | **5 years** | **6 years** |  | **2 years** | **3 years** | **4 years** | **5 years** | **6 years** |
| Natural death | Culling age | 22.39 | 18.25 | 30.82 | 34.88 | 31.50 |  | 14.69 | 11.81 | 21.12 | 23.70 | 21.09 |
|  | 6 years | 26.88 | 41.24 | 41.84 | 48.08 | 40.40 |  | 17.24 | 29.35 | 29.35 | 33.68 | 27.17 |
|  | 9 years | 34.67 | 55.00 | 57.83 | 57.65 | 59.30 |  | 23.19 | 40.00 | 42.11 | 40.79 | 42.86 |
|  | 12 years | 53.13 | 55.17 | 56.67 | 50.00 | 59.38 |  | 39.29 | 37.04 | 39.29 | 37.04 | 42.86 |
| Average |  | 34.27 | 42.41 | 46.79 | 47.65 | 47.64 |  | 23.60 | 29.55 | 32.96 | 33.80 | 33.49 |
| Structural problems | Culling age | 21.86 | 22.19 | 31.88 | 38.32 | 37.72 |  | 14.23 | 6.48 | 23.17 | 26.61 | 27.46 |
|  | 6 years | 62.38 | 50.59 | 56.82 | 68.75 | 61.96 |  | 59.18 | 37.18 | 42.50 | 51.19 | 46.99 |
|  | 9 years | 80.95 | 59.52 | 71.11 | 72.00 | 76.79 |  | 45.83 | 44.74 | 56.10 | 61.90 | 68.89 |
|  | 12 years | 85.71 | 71.43 | 71.43 | 71.43 | 77.50 |  | 66.67 | 50.00 | 50.00 | 50.00 | 61.43 |
| Average |  | 29.87 | 45.93 | 57.81 | 65.13 | 68.49 |  | 13.14 | 32.82 | 42.94 | 47.43 | 53.69 |
| Disease | Culling age | 21.44 | 13.51 | 32.60 | 37.12 | 44.22 |  | 13.15 | 3.20 | 21.39 | 32.00 | 30.36 |
|  | 6 years | 43.89 | 54.67 | 65.38 | 76.90 | 79.01 |  | 22.79 | 38.24 | 45.07 | 51.33 | 56.00 |
|  | 9 years | 85.71 | 17.24 | 50.00 | 57.67 | 65.35 |  | 9.47 | 10.71 | 34.38 | 41.05 | 49.23 |
|  | 12 years | 98.00 | 36.67 | 42.86 | 47.14 | 60.00 |  | 37.78 | 16.67 | 33.33 | 42.86 | 45.00 |
| Average |  | 55.26 | 21.27 | 47.71 | 52.21 | 59.65 |  | 21.91 | 15.60 | 33.54 | 41.81 | 47.65 |
| Fertility | Culling age | 22.22 | 13.96 | 29.47 | 36.43 | 32.94 |  | 15.10 | 7.98 | 19.94 | 24.73 | 21.78 |
|  | 6 years | 28.85 | 59.68 | 61.29 | 64.06 | 64.52 |  | 20.00 | 42.86 | 44.64 | 46.43 | 44.64 |
|  | 9 years | 9.52 | 60.71 | 70.00 | 78.13 | 91.18 |  | 7.52 | 44.00 | 51.85 | 55.56 | 55.52 |
|  | 12 years | 33.33 | 45.00 | 25.00 | 50.00 | 51.00 |  | 10.13 | 25.00 | 25.00 | 40.20 | 43.00 |
| Average |  | 35.98 | 39.84 | 46.44 | 64.66 | 67.16 |  | 26.16 | 29.96 | 35.36 | 46.68 | 47.98 |
| Performance | Culling age | 24.45 | 21.55 | 42.92 | 47.51 | 46.09 |  | 16.47 | 14.23 | 29.07 | 33.49 | 31.14 |
|  | 6 years | 28.07 | 56.72 | 59.42 | 61.43 | 61.11 |  | 20.00 | 40.32 | 41.27 | 52.94 | 42.19 |
|  | 9 years | 40.74 | 92.31 | 46.34 | 55.24 | 60.33 |  | 30.77 | 66.67 | 17.15 | 25.00 | 31.58 |
|  | 12 years | 47.00 | 54.27 | 69.23 | 73.77 | 90.00 |  | 41.43 | 47.82 | 40.00 | 60.03 | 68.18 |
| Average |  | 48.32 | 74.46 | 54.48 | 66.24 | 68.88 |  | 34.67 | 55.30 | 31.87 | 64.86 | 68.27 |
| Overall |  | 43.00 | 44.94 | 50.65 | 56.90 | 60.08 |  | 25.78 | 31.10 | 35.34 | 42.60 | 44.34 |

^1^EDL was calculated for the daughter’s average culling age and the proportion of daughters alive at 6, 9, and 12 years-old.

**Table S4.** Average difference (in percentage, %) between expected daughter longevity (EDL) from the top and bottom 1% and 10% sires, calculated for all ages at selection (i.e., from 2 to 6 years), considering all groups of culling reasons and the functional longevity definition assuming 0 after the cow was culled, and missing records when no information of calving was found at the specified age.

| **Culling reason** | **^1^EDL** | **1%** | | | | |  | **10%** | | | | |
| --- | --- | --- | --- | --- | --- | --- | --- | --- | --- | --- | --- | --- |
|  |  | **2 years** | **3 years** | **4 years** | **5 years** | **6 years** |  | **2 years** | **3 years** | **4 years** | **5 years** | **6 years** |
| Natural death | Culling age | 19.58 | 27.29 | 31.57 | 34.48 | 36.26 |  | 11.46 | 16.13 | 20.11 | 23.44 | 25.36 |
|  | 6 years | 17.98 | 40.20 | 42.19 | 48.08 | 49.04 |  | 10.71 | 25.27 | 30.11 | 33.68 | 35.42 |
|  | 9 years | 26.39 | 36.84 | 48.15 | 57.65 | 63.22 |  | 15.15 | 21.74 | 31.94 | 40.79 | 46.15 |
|  | 12 years | 48.39 | 41.38 | 44.83 | 50.00 | 58.06 |  | 29.63 | 26.92 | 26.92 | 27.04 | 32.86 |
| Average |  | 28.08 | 36.43 | 41.69 | 47.55 | 51.65 |  | 16.74 | 22.52 | 27.27 | 33.74 | 37.45 |
| Structural problems | Culling age | 18.37 | 32.54 | 36.37 | 38.28 | 39.30 |  | 12.02 | 22.15 | 24.91 | 26.69 | 27.93 |
|  | 6 years | 63.18 | 63.04 | 68.42 | 68.75 | 67.71 |  | 56.00 | 45.68 | 49.40 | 51.19 | 51.19 |
|  | 9 years | 50.00 | 59.52 | 73.91 | 82.00 | 86.79 |  | 30.77 | 43.24 | 55.00 | 61.90 | 68.18 |
|  | 12 years | 46.66 | 50.00 | 50.00 | 61.43 | 67.43 |  | 35.71 | 20.00 | 40.00 | 50.00 | 66.67 |
| Average |  | 15.39 | 51.28 | 57.18 | 65.12 | 66.31 |  | 3.27 | 32.77 | 42.33 | 47.45 | 53.49 |
| Disease | Culling age | 9.83 | 39.57 | 43.76 | 46.90 | 49.67 |  | 5.87 | 25.96 | 29.13 | 31.80 | 34.15 |
|  | 6 years | 56.82 | 77.65 | 83.53 | 85.71 | 87.21 |  | 33.33 | 55.41 | 58.67 | 61.33 | 63.16 |
|  | 9 years | 66.67 | 61.11 | 80.00 | 87.67 | 88.51 |  | 44.21 | 42.42 | 57.14 | 61.05 | 70.00 |
|  | 12 years | 88.89 | 36.67 | 33.33 | 47.14 | 58.50 |  | 62.50 | 16.67 | 16.67 | 22.86 | 27.14 |
| Average |  | 36.11 | 48.75 | 60.16 | 71.86 | 83.22 |  | 15.23 | 35.11 | 40.40 | 51.76 | 58.61 |
| Fertility | Culling age | 22.27 | 31.04 | 33.55 | 36.72 | 39.60 |  | 14.96 | 21.63 | 23.26 | 25.07 | 26.69 |
|  | 6 years | 22.00 | 52.46 | 58.06 | 64.06 | 69.23 |  | 14.58 | 38.18 | 42.86 | 45.61 | 49.12 |
|  | 9 years | 15.79 | 40.00 | 57.14 | 67.19 | 84.29 |  | 10.53 | 26.09 | 40.00 | 45.56 | 58.97 |
|  | 12 years | 25.00 | 25.00 | 60.00 | 77.07 | 80.00 |  | 18.23 | 25.00 | 25.00 | 32.11 | 30.00 |
| Average |  | 13.37 | 37.12 | 52.19 | 63.74 | 75.78 |  | 11.00 | 27.72 | 32.78 | 46.56 | 51.20 |
| Performance | Culling age | 21.06 | 40.42 | 44.06 | 46.94 | 48.70 |  | 14.03 | 28.50 | 31.17 | 33.09 | 34.41 |
|  | 6 years | 25.00 | 65.75 | 69.33 | 70.13 | 69.23 |  | 16.67 | 47.69 | 50.75 | 51.47 | 54.71 |
|  | 9 years | 28.00 | 69.70 | 83.78 | 95.24 | 92.13 |  | 16.67 | 53.33 | 62.50 | 74.29 | 81.58 |
|  | 12 years | 71.43 | 80.00 | 81.82 | 83.08 | 93.33 |  | 50.00 | 57.50 | 55.56 | 61.20 | 69.09 |
| Average |  | 36.37 | 51.47 | 69.75 | 83.85 | 88.35 |  | 24.34 | 44.26 | 49.99 | 67.21 | 69.14 |
| Overall |  | 34.90 | 47.81 | 56.19 | 63.23 | 67.74 |  | 22.94 | 32.88 | 38.55 | 44.28 | 48.91 |

^1^EDL was calculated for the daughter’s average culling age and the proportion of daughters alive at 6, 9, and 12 years-old.

**Figure S1.** Number of cows by culling age (cumulative) inside each class of culling reasons: (a) natural death, (b) structural problems, (c) disease, (d) fertility, (e) performance, and (f) miscellaneous. Age at culling equal 15 included cows culled from 15 to 20 years-old.

**Figure S2.** Number of cows by number of calvings inside each class of culling reasons: (a) natural death, (b) structural problems, (c) disease, (d) fertility, (e) performance, and (f) miscellaneous.

**Figure S3.** Proportion of codes 1 (first level; solid black patter), 0 (second level; solid white pattern) and missing (third level; dashed pattern) observed for each longevity definition over the ages. The longevity definitions are: (a) traditional longevity, (b) functional longevity assuming 0 after the cow was culled or if the cow did not have calved at the specific age, and (c) functional longevity assuming 0 only after the cow was culled, and missing records when no information of calving was found at the specific age.

**Figure S4.** Heritabilities estimated over the different ages, for all groups of culling reasons and longevity definitions, using homogeneous residual variance. The groups of culling reasons are: natural death (
), structural problems (), disease (), fertility (), performance (), miscellaneous (), and all reasons together ( ). The longevity definitions are: (a) traditional longevity, (b) functional longevity assuming 0 after the cow was culled or if the cow did not have calved at the specific age, and (c) functional longevity assuming 0 only after the cow was culled, and missing records when no information of calving was found at the specific age.

**Figure S5.** Additive genetic variance estimated over the different ages, for all groups of culling reasons and longevity definitions, using heterogeneous residual variance. The groups of culling reasons are: natural death (
), structural problems (), disease (), fertility (), performance (), miscellaneous (), and all reasons together ().The longevity definitions are: (a) traditional longevity, (b) functional longevity assuming 0 after the cow was culled or if the cow did not have calved at the specific age, and (c) functional longevity assuming 0 only after the cow was culled, and missing records when no information of calving was found at the specific age.

**Figure S6.** Permanent environmental variance estimated over the different ages, for all groups of culling reasons and longevity definitions, using heterogeneous residual variance. The groups of culling reasons are: natural death (
), structural problems (), disease (), fertility (), performance (), miscellaneous (), and all reasons together ( ). The longevity definitions are: (a) traditional longevity, (b) functional longevity assuming 0 after the cow was culled or if the cow did not have calved at the specific age, and (c) functional longevity assuming 0 only after the cow was culled, and missing records when no information of calving was found at the specific age.

**Figure S7.** Herd-year-season variance estimated over the different ages, for all groups of culling reasons and longevity definitions, using heterogeneous residual variance. The groups of culling reasons are: natural death (
), structural problems (), disease (), fertility (), performance (), miscellaneous (), and all reasons together ( ). The longevity definitions are: (a) traditional longevity, (b) functional longevity assuming 0 after the cow was culled or if the cow did not have calved at the specific age, and (c) functional longevity assuming 0 only after the cow was culled, and missing records when no information of calving was found at the specific age.

**Figure S8.** Genetic correlations estimated between the different longevity definitions over ages, for all groups of known culling reasons. The groups of culling reasons are: natural death (a, b, c); structural problems (d, e, f); disease (g, h, i); fertility (j, k, l); and performance (m, n, o). The longevity definitions are: traditional longevity (TL); functional longevity assuming 0 after the cow was culled or if the cow did not have calved at the specific age (FL_a_); and functional longevity assuming 0 only after the cow was culled, and missing records when no information of calving was found at the specific age (FL_b_), respectively.

**Figure S9.** Prediction accuracy of the daughter’s average culling age calculated inside each group of known culling reasons and longevity definitions. The groups of culling reasons are: natural death (a); structural problems (b); disease (c); fertility (d); and performance (e). The longevity definitions are: traditional longevity (TL); functional longevity assuming 0 after the cow was culled or if the cow did not have calved at the specific age (FL_a_); and functional longevity assuming 0 only after the cow was culled, and missing records when no information of calving was found at the specific age (FL_b_), respectively.

**Figure S10.** Prediction accuracy of the expected daughter longevity (EDL) calculated inside each group of known culling reasons and longevity definitions. The EDL was calculated for the proportion of daughters alive at 6, 9, and 12 years-old. The groups of culling reasons are: natural death (a, b, c); structural problems (d, e, f); disease (g, h, i); fertility (j, k, l); and performance (m, n, o). The longevity definitions are: traditional longevity (TL); functional longevity assuming 0 after the cow was culled or if the cow did not have calved at the specific age (FL_a_); and functional longevity assuming 0 only after the cow was culled, and missing records when no information of calving was found at the specific age (FL_b_), respectively.
